# Independent and combined effects of improved water, sanitation, and hygiene, and improved complementary feeding, on stunting and anaemia among HIV-exposed children in rural Zimbabwe: a cluster-randomised controlled trial

**DOI:** 10.1016/S2352-4642(18)30340-7

**Published:** 2019-02

**Authors:** Andrew J Prendergast, Bernard Chasekwa, Ceri Evans, Kuda Mutasa, Mduduzi N N Mbuya, Rebecca J Stoltzfus, Laura E Smith, Florence D Majo, Naume V Tavengwa, Batsirai Mutasa, Goldberg T Mangwadu, Cynthia M Chasokela, Ancikaria Chigumira, Lawrence H Moulton, Robert Ntozini, Jean H Humphrey

**Affiliations:** a Zvitambo Institute for Maternal and Child Health Research, Harare, Zimbabwe; b Blizard Institute, Queen Mary University of London, London, UK; c Department of International Health, Johns Hopkins Bloomberg School of Public Health, Baltimore MD, USA; d Global Alliance for Improved Nutrition, Washington DC, USA; e Division of Nutritional Sciences, Cornell University, Ithaca, NY, USA; f Department of Epidemiology and Environmental Health, School of Public Health and Health Professions, University at Buffalo, Buffalo, NY, USA; g Ministry of Health and Child Care, Government of Zimbabwe, Harare, Zimbabwe

## Abstract

**Background:**

Children exposed to HIV have a high prevalence of stunting and anaemia. We aimed to test the effect of improved infant and young child feeding (IYCF) and improved water, sanitation, and hygiene (WASH) on child linear growth and haemoglobin concentrations.

**Methods:**

We did a cluster randomised 2 × 2 factorial trial in two districts in rural Zimbabwe. Women were eligible for inclusion if they permanently lived in the trial clusters (ie, the catchment area of between one and four village health workers employed by the Zimbabwean Ministry of Health and Child Care) and were confirmed pregnant. Clusters were randomly allocated to standard of care (52 clusters); IYCF (20 g small-quantity lipid-based nutrient supplement daily for infants from 6 months to 18 months, complementary feeding counselling with context-specific messages, longitudinal delivery, and reinforcement; 53 clusters); WASH (ventilated, improved pit latrine, two hand-washing stations, liquid soap, chlorine, play space, and hygiene counselling; 53 clusters); or IYCF plus WASH (53 clusters). Participants and fieldworkers were not masked. Our co-primary outcomes were length for age Z score and haemoglobin in infants at 18 months of age. Here, we report these outcomes in the HIV-exposed children, analysed by intention to treat. We estimated the effects of the interventions by comparing the two IYCF groups with the two non-IYCF groups and the two WASH groups with the two non-WASH groups, except for outcomes with an important statistical interaction between the interventions. The trial is registered at ClinicalTrials.gov (NCT01824940) and is now complete.

**Findings:**

Between Nov 22, 2012, and March 27, 2015, 726 HIV-positive pregnant women were included in the trial. 668 children were evaluated at 18 months (147 from 46 standard of care clusters; 147 from 48 IYCF clusters; 184 from 44 WASH clusters; 190 from 47 IYCF plus WASH clusters). Of the 668 children, 22 (3%) were HIV-positive, 594 (89%) HIV-exposed uninfected, and 52 (8%) HIV-unknown. The IYCF intervention increased mean length for age Z score by 0·26 (95% CI 0·09–0·43; p=0·003) and haemoglobin concentration by 2·9 g/L (95% CI 0·90–4·90; p=0·005). 165 (50%) of 329 children in the non-IYCF groups were stunted, compared with 136 (40%) of 336 in the IYCF groups (absolute difference 10%, 95% CI 2–17); and the prevalence of anaemia was also lower in the IYCF groups (45 [14%] of 319) than in the non-IYCF groups (24 [7%] of 329; absolute difference 7%, 95% CI 2–12). The WASH intervention had no effect on length or haemoglobin concentration. There were no trial-related adverse or serious adverse events.

**Interpretation:**

Since HIV-exposed children are particularly vulnerable to undernutrition and responded well to improved complementary feeding, IYCF interventions could have considerable benefits in areas of high antenatal HIV prevalence. However, elementary WASH interventions did not lead to improvements in growth.

**Funding:**

Bill & Melinda Gates Foundation, UK Aid, Wellcome Trust, Swiss Development Cooperation, US National Institutes of Health, and UNICEF.

## Introduction

Perinatal HIV infection is a major risk factor for child undernutrition, but programmes to prevent mother-to-child transmission have reduced new infant infections by almost half since 2010.^1^ However, there is an increasing population of HIV-exposed uninfected children,^2^ who have more undernutrition than HIV-unexposed children. In a birth cohort of 14 110 Zimbabwean infants, HIV-exposed uninfected children had 23% increased odds of stunting and 56% increased odds of wasting at 12 months of age,^3^ and mean head circumference was lower throughout the first year of life compared with HIV-unexposed infants.^4^ Infants born to HIV-positive mothers are therefore vulnerable to undernutrition, regardless of their own infection status, and targeted interventions to promote healthy growth are required.

**Research in context**

**Evidence before this study**

In low-income countries, child stunting is highly prevalent, particularly in HIV-exposed uninfected children compared with HIV-unexposed children. We searched PubMed with the following search terms: (stunting OR length OR height OR haemoglobin OR hemoglobin OR anaemia OR anemia) AND (child OR infant) AND (feeding OR WASH OR water OR sanitation OR hygiene) AND (HIV) from database inception up to June 1, 2018 with no language restrictions. An observational study from Tanzania reported less stunting among HIV-exposed children with higher infant and child feeding index scores, and observational data from the Côte d'Ivoire showed that improved complementary feeding at age 6 months was associated with better linear growth and a lower prevalence of stunting during the subsequent 12 months. A non-controlled intervention of nutritional support for non-breastfed, HIV-exposed Haitian children found a reduction in stunting compared with an historical control population at 6 months and 12 months of age. In trial data of maternal nutritional supplementation for breastfeeding mothers, there was no effect on linear growth among HIV-exposed children in South Africa or Malawi. Modest improvements in linear growth of HIV-exposed children have been shown in trials of improved infant feeding among infants breastfed for only short periods (<6 months) or not at all. Provision of lipid-based nutrient supplements instead of breastfeeding from 6 months of age improved linear growth in Zambian HIV-exposed children. In formula-fed, HIV-exposed uninfected children in the USA, Bahamas, and Brazil, more concentrated formula improved weight compared with standard formula. We found one study assessing the effect of nutrition on haemoglobin in HIV-exposed infants: in Zambia, rich fortification of porridge increased haemoglobin concentration and reduced anaemia. We did not identify any studies of WASH or combined WASH and complementary feeding on linear growth of HIV-exposed children. We did not identify any studies investigating the relationship between WASH and haemoglobin concentration or anaemia.

**Added value of this study**

To our knowledge, this is the first randomised trial to investigate the effect of improved water, sanitation, and hygiene (WASH) and improved infant and young child feeding (IYCF)—independently and in combination—in HIV-exposed children. This study was done in a rural African setting with high antenatal HIV prevalence, and high rates of child stunting and anaemia. IYCF and WASH interventions were informed by extensive formative research, were culturally appropriate, and led to significant behaviour change. Consistent with previous studies, we found that complementary feeding improved linear growth and haemoglobin concentration in HIV-exposed children, reducing stunting by 20% and anaemia by 50%. We found no effect on linear growth or haemoglobin concentration with the WASH intervention.

**Implications of all the available evidence**

The findings of this trial, supported by previous studies in low-income and middle-income countries, show that IYCF modestly improves linear growth and haemoglobin concentrations in HIV-exposed children, and would lead to substantial reductions in stunting and anaemia if delivered at scale. Because HIV-exposed children are particularly vulnerable to undernutrition, populations with a high maternal HIV prevalence may particularly benefit from complementary feeding interventions. However, combining complementary feeding with the elementary household-level WASH interventions commonly implemented in rural areas of low-income and middle-income countries (ie, pit latrines, hand-washing stations not connected to a water source, and point-of-use chlorination of drinking water with monthly behaviour-change promotion) provides no additional benefit compared with IYCF alone, indicating that more effective WASH interventions are required.

Stunting, the most prevalent form of undernutrition, is associated with increased child mortality^5^ and reduced school attainment, and perpetuates an intergenerational cycle of inequity.^6^ Anaemia often co-exists with stunting, is similarly intractable,^7^ and is another major cause of impaired neurodevelopment.^8^ Since optimising infant and young child feeding (IYCF) only modestly improves linear growth,^9^ there is an increasing recognition that multisectoral approaches are required to tackle stunting. Improving water, sanitation, and hygiene (WASH) might affect growth and anaemia by reducing diarrhoeal disease and preventing environmental enteric dysfunction, a subclinical inflammatory disorder of the small intestine that is highly prevalent among children living in poverty.^10^

The Sanitation Hygiene Infant Nutrition Efficacy (SHINE) trial was designed to test the independent and combined effects of improved IYCF and improved WASH, on both stunting and anaemia in an area of high antenatal HIV prevalence in rural Zimbabwe. We have previously reported trial results of children born to HIV-negative women;^11^ here, we report the effect of IYCF and WASH on stunting and anaemia among HIV-exposed children.

## Methods

### Study design and participants

The SHINE trial design has been reported previously;^10^, ^11^ the protocol and statistical analysis plan are available online. Briefly, SHINE was a cluster-randomised community-based 2 × 2 factorial trial done in two contiguous rural districts in Zimbabwe with 15% antenatal HIV prevalence. Clusters were defined as the catchment area of 1–4 village health workers employed by the Ministry of Health and Child Care.

Village health workers did prospective pregnancy surveillance and established date of last menstrual period among pregnant women, and referred pregnant women to SHINE research nurses for trial enrolment. Women were eligible if they permanently resided in a study cluster and were confirmed pregnant. Over the recruitment period, the cutoff of gestational age for recruitment eligibility was gradually liberalised to maximise recruitment (appendix). The Medical Research Council of Zimbabwe and the Institutional Review Board of the Johns Hopkins Bloomberg School of Public Health approved the study protocol. All participants provided written informed consent.

### Randomisation and masking

Clusters were allocated (1:1:1:1) to one of four treatment groups: standard of care, IYCF, WASH, or IYCF plus WASH at a public event. A highly constrained randomisation technique achieved balance across groups for 14 variables related to geography, demography, water access, and sanitation coverage (appendix).^12^ Masking of participants and fieldworkers was not possible, but investigators analysing the data were masked to group allocation.

### Procedures

Interventions were informed by extensive formative research and piloting.^10^ All women were scheduled to receive 15 modules delivered by group-specific village health workers (VHWs), with behaviour-change messages and interactive tools between enrolment and 12 months postnatal (approximately one visit per month); other family members were encouraged to participate. At each visit, previous information was reviewed before introducing new information to create a sequenced integrated longitudinal intervention. Between 13 and 17 months, VHWs visited monthly, providing routine care and delivering intervention supplies; during these visits VHWs encouraged participants to practise relevant behaviours, although structured modules were not implemented (see key messages and supplies in the appendix, and lesson plans and interactive tools online).

Standard of care messages comprised promotion of exclusive breastfeeding to 6 months, uptake of antenatal and neonatal care, prevention of mother-to-child HIV transmission, immunisations, family planning, and standard IYCF information based on WHO recommend-ations. Groups with the IYCF component received all standard of care messages plus information about the importance of nutrition for infant health, growth, and development; feeding nutrient-dense food and a 20 g small-quantity lipid-based nutrient supplement (SQ-LNS; Nutriset, Malaumay, France) daily from age 6 months to 18 months; processing locally available foods to facilitate mastication and swallowing; feeding during illness; and dietary diversity. The IYCF modules therefore addressed specific contextual barriers through a sequential longitudinal intervention based on successive messages and reinforcement. VHWs also made monthly deliveries of 30 20 g sachets of small-quantity lipid-based nutrient supplement from infant age 6 months through to 18 months. The WASH component included all standard of care messages plus information about safe disposal of faeces; hand-washing with soap after faecal contact and before preparing food, eating food or feeding children; protection of infants from geophagia and animal faeces ingestion; chlorination of drinking water; and hygienic preparation of complementary food. Additionally, a ventilated improved pit latrine was provided within 6 weeks of enrolment; two hand-washing stations, plastic mat and play yard (North States, Minneapolis, MN, USA), and monthly delivery of soap and chlorine (WaterGuard, Nelspot, Zimbabwe) were provided. A latrine was constructed in the non-WASH groups following trial completion.

Research nurses made home visits at baseline (approximately 2 weeks after consent), 32 weeks' gestation, and at 1 month, 3 months, 6 months, 12 months, and 18 months post partum to assess maternal and household characteristics and trial outcomes. At baseline, mothers had height, weight, and mid-upper arm circumference measured, and were tested for haemoglobin concentrations (Hemocue, Ängelholm, Sweden), *Schistosoma haematobium* infection (by urinary microscopy), and HIV. HIV-positive women were encouraged to seek immediate antenatal care to prevent mother-to-child transmission. Other maternal and household characteristics were assessed, including dietary diversity, food insecurity, household wealth, and maternal capabilities.^13^

Infant birth date, weight, and delivery details were transcribed from health facility records. The trial provided Tanita BD-590 infant scales (Weigh & Measure, Olney, MD, USA) to all health institutions in the study area and trained facility staff. Gestational age at delivery was calculated from last menstrual period dates. At the 18-month postnatal visit (trial endpoint), mothers and infants were visited anywhere in the country for the intention-to-treat analyses of primary outcomes; however, given the household-based nature of the interventions, intermediate visits were done only when the mother was available in the household where she consented. At 18 months postnatal, infant point-of-care haemoglobin concentration was measured (HemoCue, Ängelholm, Sweden). Infant length was calculated as the median of three measurements; weight, head circumference, and mid-upper arm circumference were also measured (appendix). Infant diarrhoea (three or more loose or watery stools in 24 h), dysentery (stool with blood or mucus), and acute respiratory infection (fast or difficult breathing) were assessed by 7 day maternal recall at postnatal visits. Infants with acute malnutrition or illness were referred to clinics.

Adverse events and serious adverse events were ascertained by research nurses during visits, and by village health workers during intervention delivery contacts, and reported to a senior research nurse who collected details. Events were reviewed by the study physician (AJP) to determine relatedness to trial interventions before reporting to the responsible institutional review boards. An independent data safety and monitoring board comprising two physicians from Zimbabwe and a statistician from the UK reviewed interim adverse event data.

Mothers were tested for HIV status at baseline with a rapid test algorithm (Determine HIV 1/2 test [Alere International Limited, Ballybrit, Ireland], followed by INSTI HIV 1/2 test [bioLytical Laboratories Inc., Richmond, BC, Canada] if positive). HIV-positive women had CD4 counts measured (Pima Analyser [Alere International Limited, Ballybrit, Ireland]) and were referred to local clinics. Viral load was not measured in the trial. National guidelines for prevention of mother-to-child HIV transmission changed from WHO Option B (maternal antiretrovial therapy [ART] from 14 gestational weeks until the end of breastfeeding) to Option B+ (lifelong ART for all pregnant and breastfeeding women) in November, 2013. Women were encouraged to initiate co-trimoxazole and ART, to exclusively breastfeed, and to attend clinic at 6 weeks post partum for early infant diagnosis and initiation of infant co-trimoxazole. Women testing HIV-negative at baseline were retested at 32 gestational weeks and 18 months post partum to detect sero-conversion.

HIV-positive mothers were invited to enrol in a substudy, in which infant blood was collected at 1 month, 3 months, 6 months, 12 months, and 18 months and tested for HIV; infants of mothers declining substudy enrolment were only tested at 18 months. Children were classified as HIV-positive or HIV-exposed uninfected based on results at 18 months, or their last available test. Children not tested at 18 months owing to caregiver refusal, defaulted visits, or loss to follow-up were classified as HIV-unknown. Inconclusive or discordant results were retested to confirm status; if no further samples were available or repeat testing was inconclusive, children were classified as HIV-unknown. Before 18 months of age, HIV was diagnosed by DNA PCR on dried blood-spot samples or RNA PCR on plasma; and after 18 months, by PCR or rapid test algorithm, depending on samples provided. All HIV-positive children were referred for ART initiation.

### Outcomes

The coprimary outcomes were length for age Z score and haemoglobin concentration in infants at age 18 months (allowable age range 76–130 weeks). Secondary outcomes were stunting (length for age Z score <–2), severe stunting (length for age Z score <–3), anaemia (haemoglobin <105 g/L), severe anaemia (<70 g/L); weight for age Z score, underweight (weight for age Z score <–2), weight for length Z score, wasting (weight for length Z score <–2), mid-upper arm circumference for age Z score and head circumference for age Z score at 18 months; 7-day maternal recall of diarrhoea, dysentery, and acute respiratory infection at 12 months and 18 months; and all-cause mortality up to 18 months. Intervention uptake was assessed at all visits and reported here for the 12-month visit.

### Statistical analysis

Sample size calculation was done for HIV-unexposed infants.^11^ No specific sample size calculation for outcomes among HIV-exposed infants was done. All analyses were done on an intention-to-treat basis at the child level.

For primary analyses, we used generalised estimating equations that accounted for within-cluster correlation and contained two dummy variables representing the main effect of the IYCF intervention (the two IYCF-containing groups compared to the two groups without IYCF) and the WASH intervention (the two WASH-containing groups compared to the two groups without WASH), unadjusted for other covariates, with an exchangeable working correlation structure.^11^ Although the study was not powered to detect a statistical interaction between the IYCF and WASH interventions, we estimated these interactions for each outcome. When the interaction was significant (ie, p<0·05 according to the Wald test) or had a sizeable point estimate (ie, RR >2 or <0·5 when comparing ratio-of-ratios, or difference-of-differences >0·25 SDs when comparing continuous outcomes), results are based on a regression model with three dummy variables to represent IYCF, WASH and IYCF plus WASH compared to standard of care instead of the model of two terms. In adjusted analyses we controlled for prespecified baseline covariates, which were initially assessed in bivariate analyses to identify those with an important association with the outcome (ie, p<0·2 or RR >2·0 or <0·5 for dichotomous outcomes, and p<0·2 or difference >0·25 SDs for continuous outcomes). Selected covariates were entered in a multivariable regression model; a forward stepwise selection procedure was implemented with p<0·2 to enter. A log-binomial specification was used to facilitate estimation of relative risks (RR). Depending on the analysis, other methods for comparing groups while accounting for within-cluster correlation included multinomial and ordinal regression models with robust variance estimation, and Somers' D for medians.

In a per-protocol analysis, we examined the effect of the interventions when behaviour-change modules were delivered at high fidelity (which was predefined for the IYCF plus WASH group as receiving all ten core modules and for the other study groups as receiving all modules scheduled at the same timepoints when IYCF plus WASH core modules were delivered). A prespecified subgroup analysis of primary outcomes by infant sex was planned if the interaction terms were significant (p<0·05). A sensitivity analysis excluded children testing HIV-positive or HIV-unknown at 18 months.

We used Stata (version 14) for all analyses. The study is registered with ClinicalTrials.gov, number NCT01824940.

### Role of the funding source

The funders of the study had no role in study design, data collection, data analysis, data interpretation, or writing of the report. The corresponding author had full access to all the data in the study and had final responsibility for the decision to submit for publication.

## Results

Between Nov 22, 2012, and March 27, 2015, 5280 pregnant women were enrolled from 211 clusters at a median gestational age of 12 (IQR 9–16) weeks (figure). Among 4843 livebirths, 738 infants were born to 726 mothers testing HIV-positive during pregnancy and are included in this analysis. During the postnatal period, 51 (7%) of 738 infants died, of whom three were HIV-positive, two HIV-exposed uninfected, and 46 HIV-unknown.

**Figure:**
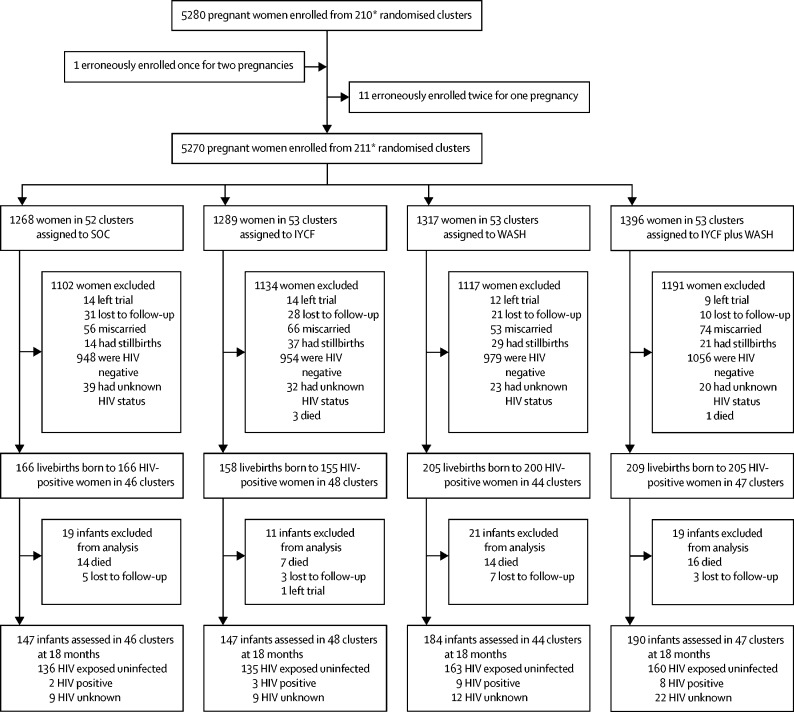
Trial profile SOC=standard of care. IYCF=infant and young child feeding. WASH=water, sanitation, and hygiene. *212 clusters were randomly assigned, 53 in each of the four trial groups. After randomisation, one cluster was excluded because it was in an urban area, one was excluded because the village health worker covering it mainly had clients outside the study area, and two more were merged on the basis of subsequent data for village health worker coverage. Three new cluster designations were created because of anomalies in the original mapping. For two of these cluster, the trial group was clear; the third contained areas that were in two trial groups, and was assigned to the under-represented group, resulting in 53 clusters in each group. All these changes occurred before enrolment began. When enrolment was completed, however, no women were enrolled in one cluster in the SOC group and thus 211 clusters were available for analysis.

Among households of HIV-positive women at baseline, one-third had a latrine, two-thirds had some solar powered electricity, and around half of all household members practised open defecation (table 1). Water access was poor. Most mothers were married and had completed on average 9 years of schooling but few had employment. Nutritional status was generally good; no women had mid-upper arm circumference less than 19 cm. Mean CD4 count at baseline was 473 cells/μL (SD 221); most mothers received ART and just over half received co-trimoxazole during pregnancy (table 1). There were some minor baseline imbalances between groups. Women in the standard-of-care group were slightly poorer, with a lower wealth index and higher proportion of homes with no electricity and unimproved floors. More households in the IYCF plus WASH and WASH groups had a hand-washing station at baseline. Women in the WASH and IYCF plus WASH groups had slightly lower mean CD4 counts; mothers in the WASH group were less likely to be taking ART but more likely to be taking co-trimoxazole. Infant birth characteristics were similar across treatment groups. The majority were born by vaginal delivery and had institutional deliveries. Mean birthweight was 2·99 (SD 0·50) kg.

**Table 1:** Maternal, household, and infant baseline characteristics of HIV-positive mothers and their liveborn infants

				**Standard of care**	**IYCF**	**WASH**	**IYCF plus WASH**
Mothers				166	155	200	205
Infants				166	158	205	209
Mothers completing baseline visit				164	151	199	203
Household characteristics							
	Median number of occupants (IQR)			4 (3–5)	4 (3–6)	5 (3–6)	4 (3–6)
	Wealth quintile^14^						
		Lowest		46/163 (28%)	39/151 (26%)	50/197 (25%)	56/201 (28%)
		Second		47/163 (29%)	27/151 (18%)	44/197 (22%)	49/201 (24%)
		Middle		32/163 (20%)	31/151 (21%)	39/197 (20%)	34/201 (17%)
		Fourth		18/163 (11%)	35/151 (23%)	27/197 (14%)	26/201 (13%)
		Highest		20/163 (12%)	19/151 (13%)	37/197 (19%)	36/201 (18%)
	Electricity						
		Power grid		5/163 (3%)	3/151 (2%)	4/197 (2%)	7/201 (4%)
		Other power source					
			Generator	4/163 (3%)	7/151 (5%)	2/197 (1%)	5/202 (3%)
			Solar	96/163 (59%)	102/151 (68%)	127/197 (65%)	128/202 (64%)
		No electricity		63/163 (39%)	42/151 (28%)	68/197 (35%)	69/202 (34%)
	Sanitation						
		Household members who openly defecate		366/609 (60%)	349/621 (56%)	392/788 (50%)	402/780 (52%)
		Any latrine at household		46/163 (28%)	56/147 (38%)	82/196 (42%)	74/197 (38%)
		Improved latrine at household		38/163 (23%)	48/147 (33%)	74/196 (38%)	66/196 (34%)
		Improved latrine with well-trodden path and not shared		21/157 (13%)	37/144 (26%)	53/193 (28%)	39/188 (21%)
	Water						
		Main source of household drinking water is improved		101/163 (62%)	88/147 (60%)	114/194 (59%)	119/197 (60%)
		Treat drinking water to make it safer		20/160 (13%)	20/144 (14%)	20/192 (10%)	24/197 (12%)
		Median one-way walk time to fetch water (IQR), min		10 (5–20)	10 (3–15)	10 (5–20)	10 (5–20)
		Mean per-capita water volume collected in past 24 h (SD), L		8·7 (3·7) [n=125]	9·9 (7·6) [n=121]	9·5 (5·9) [n=160]	9·7 (7·6) [n=169]
	Hygiene						
		Handwashing station at household		4/141 (3%)	8/142 (6%)	32/188 (17%)	24/187 (13%)
		Handwashing station with water and rubbing agent		0/141	0/138	0/187	1/187 (<1%)
		Improved floor*		66/162 (41%)	75/150 (50%)	97/194 (50%)	99/196 (51%)
		Median number of chickens (IQR)		4 (0–9)	4 (0–10)	5 (2–9)	4 (0–8)
		Livestock observed inside home		64/164 (39%)	45/152 (30%)	71/196 (36%)	63/199 (32%)
		Faeces observed in yard		58/162 (36%)	42/152 (28%)	62/194 (32%)	47/198 (24%)
	Diet quality and food security						
		Household meets minimum dietary diversity^15^		51/137 (37%)	53/130 (41%)	62/174 (36%)	70/171 (41%)
		Median coping strategies Index (IQR)^16^		3 (0–10)	2 (0–13)	2 (0–9·5)	3 (0–11)
Maternal characteristics							
	Mean age (SD), years			29·7 (6·0)	29·0 (6·8)	28·4 (4·8)	29·6 (5·7)
	Mean height (SD), cm			161·1 (7·2)	160·0 (5·8)	160·5 (6·9)	159·4 (6·5)
	Mean mid-upper-arm circumference (SD), cm			26·4 (3·4)	26·1 (3·0)	26·4 (2·1)	26·1 (2·6)
	Positive microscopy for *Schistosoma haematobium*			16/154 (10%)	10/148 (7%)	24/185 (13%)	21/192 (11%)
	Mean years of completed schooling (SD)			9·4 (1·8)	9·0 (2·2)	8·9 (2·3)	9·1 (2·4)
	Median parity (IQR)			2 (1–3) [n=113]	2 (1–3) [n=110]	2 (1–3) [n=133]	2 (1–3) [n=160]
	Married			148/156 (95%)	140/149 (94%)	178/188 (95%)	177/189 (94%)
	Employed			17/160 (11%)	16/151 (11%)	22/197 (11%)	12/202 (6%)
	Religion						
		Apostolic		77/158 (49%)	67/149 (45%)	88/189 (47%)	98/193 (51%)
		Other Christian religions		66/158 (42%)	67/149 (45%)	77/189 (41%)	78/193 (40%)
		Other non-Christian religions		15/158 (10%)	15/149 (10%)	24/189 (13%)	17/193 (9%)
	HIV disease severity and treatment						
		Mean CD4 count in pregnancy (SD), cells per μL†		503 (215) [n=129]	496 (191) [n=136]	444 (184) [n=163]	464 (186) [n=175]
		Antiretroviral therapy during pregnancy‡		139/166 (84%)	130/155 (84%)	151/200 (76%)	167/205 (82%)
		Co-trimoxazole prophylaxis during pregnancy§		84/166 (51%)	84/155 (54%)	123/200 (62%)	111/205 (54%)
Infant characteristics							
	Sex						
		Female		80/165 (48%)	77/156 (49%)	97/205 (47%)	113/207 (55%)
		Male		85/165 (52%)	79/156 (51%)	108/205 (53%)	94/207 (45%)
	Mean birthweight (SD), kg			3·00 (0·50)	2·99 (0·41)	3·03 (0·67)	2·99 (0.59)
	Birthweight <2500 g			20/166 (12%)	18/158 (11%)	23/205 (11%)	23/209 (11%)
	Institutional delivery			119/144 (83%)	119/142 (84%)	148/176 (84%)	149/176 (85%)
	Vaginal delivery			139/147 (95%)	131/141 (93%)	165/179 (92%)	166/183 (91%)

IYCF=infant and young child feeding. WASH=water, sanitation, and hygiene. Baseline for mothers was 2 weeks after consent (approximately 14 weeks gestation). Baseline for infants was at birth. Values are n (%), unless stated.

* Improved floor defined as concrete, brick, cement, or tile. Unimproved floor defined as mud, earth, sand, or dung.

† CD4 count at baseline visit, or at 32 gestational week visit if no baseline result.

‡ Includes any exposure to antiretroviral therapy during pregnancy.

§ Includes any exposure to co-trimoxazole during pregnancy.

Fidelity of intervention delivery by the trial was high (table 2). Among WASH households, almost all received latrines and handwashing stations, the vast majority received a play mat and yard, and receipt of soap and chlorine was high. Among IYCF households, receipt of SQ-LNS was similarly high. Across all groups, VHWs completed the vast majority of planned intervention visits, although module delivery was slightly higher in the IYCF groups than in the non-IYCF groups (table 2). Intervention uptake was assessed by participant behaviours at the 12-month postnatal visit, when three-quarters of women were in their primary home and available for the visit (table 2). Women assessed at 12 months were, on average, 3 years older, with similar parity, more likely to be married, and less likely to be employed at baseline compared with women not assessed at 12 months. Baseline indicators of diet, water, sanitation, and hygiene were similar between women who were and were not assessed for intervention uptake at 12 months (appendix).

**Table 2:** Intervention delivery and participant uptake by treatment group

		**Data source**	**Standard of care**	**IYCF**	**WASH**	**IYCF plus WASH**	Combined WASH*	Non-WASH*	**p value**	**Combined IYCF**†	**Non-IYCF**†	**p value**
**Intervention delivery at baseline**												
Children with 18-month outcomes (on whom inferences are based), n		Trial logs	147	147	184	190	374	294	..	337	331	..
WASH supplies												
	SHINE-installed ventilated improved pit latrine	Trial logs	NA	NA	181/184 (98%)	188/190 (99%)	369/374 (99%)	NA	..	NA	NA	..
	Two handwashing stations (ie, Tippy Taps) delivered	Trial logs	NA	NA	184/184 (100%)	190/190 (100%)	374/374 (100%)	NA	..	NA	NA	..
	Baby mat delivered	Trial logs	NA	NA	174/184 (95%)	183/190 (96%)	358/374 (96%)	NA	..	NA	NA	..
	Play yard delivered	Trial logs	NA	NA	171/184 (92%)	180/190 (95%)	351/374 (94%)	NA	..	NA	NA	..
	Median liquid soap deliveries (IQR)‡	Trial logs	NA	NA	19 (18–20)	20 (18–20)	20 (18–20)	NA	..	NA	NA	..
	Received at least 16 (80% of expected) soap deliveries	Trial logs	NA	NA	145/184 (79%)	159/190 (84%)	304/374 (81%)	NA	..	NA	NA	..
	Median WaterGuard deliveries (IQR)‡	Trial logs	NA	NA	15 (14–15)	15 (15–15)	15 (14–15)	NA	..	NA	NA	..
	Received at least 12 (80% of expected) WaterGuard deliveries	Trial logs	NA	NA	150/184 (82%)	159/190 (84%)	309/374 (83%)	NA	..	NA	NA	..
IYCF supplies												
	Median SQ-LNS deliveries (IQR)	Trial logs	NA	13 (12–13)	NA	13 (13–13)	NA	NA	..	13 (12–13)	NA	..
	Received ≥11 (80% of expected) SQ-LNS deliveries	Trial logs	NA	117/147 (80%)	NA	157/190 (83%)	NA	NA	..	274/337 (81%)	NA	..
Behaviour change modules												
	Median intervention modules (IQR)	VHW report	15 (11–15)	15 (14–15)	15 (13·5–15)	15 (14–15)	15 (14–15)	15 (13–15)	0·58	15 (14–15)	15 (13–15)	0·003
	Percent intervention modules completed (% due)	VHW report	2349/2748 (86%)	3392/3651 (93%)	4243/4713 (90%)	5214/5657 (92%)	9457/10370 (91%)	5741/6399 (90%)	0·42	8606/9308 (93%)	6592/7461 (88%)	0·025
**Participant behaviours at 12 month visit**												
Number of mothers with 12 month and 18 month outcomes		Trial logs	113	119	131	158	289	232	..	277	244	..
Number of children with 12 month and 18 month outcomes		Trial logs	113	122	135	162	297	235	..	284	248	..
WASH behaviours												
	Household members who practice open defecation	Maternal report	172/320 (54%)	183/442 (41%)	3/546 (1%)	0/699	3/1245 (<1%)	355/762 (47%)	<0·001	NA	NA	..
	Any latrine at household	Observation	26/110 (24%)	45/120 (38%)	131/131 (100%)	155/155 (100%)	286/286 (100%)	71/230 (31%)	<0·001	NA	NA	..
	Improved latrine at household	Observation	24/110 (22%)	33/120 (28%)	131/131 (100%)	154/154 (100%)	285/285 (100%)	57/230 (25%)	<0·001	NA	NA	..
	Improved latrine at household with well-trodden path, not used for storage, and not shared with other households	Observation and maternal report	19/110 (17%)	23/120 (19%)	113/131 (86%)	134/154 (87%)	247/285 (87%)	42/230 (18%)	<0·001	NA	NA	..
	Handwashing station at household	Observation	2/103 (2%)	7/117 (6%)	133/133 (100%)	157/158 (100%)	290/291 (100%)	9/220 (4%)	<0·001	NA	NA	..
	Handwashing station with water and rubbing agent at household	Observation	1/102 (1%)	2/114 (2%)	101/122 (83%)	116/139 (84%)	217/261 (83%)	3/216 (1%)	<0·001	NA	NA	..
	Ever treats drinking water to make it safer	Maternal report	9/109 (8%)	20/120 (17%)	107/130 (82%)	138/157 (85%)	245/287 (85%)	29/229 (13%)	<0·001	NA	NA	..
	Disposes rinse water from cleaning infant nappies with faeces in a latrine	Maternal report	24/109 (22%)	37/115 (32%)	107/132 (81%)	114/145 (79%)	221/277 (80%)	61/224 (27%)	<0·001	NA	NA	..
	Play space is visibly clean	Observation	NA	NA	117/127 (92%)	134/150 (89%)	251/277 (91%)	NA	..	NA	NA	..
	Child ever observed to eat soil	Maternal report	80/109 (73%)	75/121 (62%)	39/132 (30%)	26/154 (17%)	65/286 (23%)	155/230 (67%)	<0·001	NA	NA	..
	Child ever observed to eat chicken faeces	Maternal report	21/109 (19%)	18/121 (15%)	3/132 (2%)	6/153 (4%)	9/285 (3%)	39/230 (17%)	<0·001	NA	NA	..
IYCF behaviours												
	Child is still breastfeeding	Maternal report	100/112 (89%)	107/121 (88%)	119/134 (89%)	144/157 (92%)	NA	NA	..	251/278 (90%)	219/246 (89%)	0·66
	Mother reports correct ways to feed child during and after illness	Maternal report	83/112 (74%)	83/120 (69%)	92/132 (70%)	109/155 (70%)	NA	NA	..	192/275 (70%)	175/244 (72%)	0·65
	Infant diet met minimum dietary diversity in past 24 h§	Maternal report	60/104 (58%)	73/119 (61%)	59/122 (48%)	106/146 (73%)	NA	NA	..	179/265 (68%)	119/226 (53%)	0·002
	Infant consumed iron rich food in the past 24 h§	Maternal report	64/110 (58%)	117/120 (98%)	61/131 (47%)	146/155 (94%)	NA	NA	..	263/275 (96%)	125/241 (52%)	<0·001
	Infant consumed animal source food in the past 24 h§	Maternal report	78/110 (71%)	87/120 (73%)	78/130 (60%)	114/152 (75%)	NA	NA	..	201/272 (74%)	156/240 (65%)	0·048
	Infant consumed vitamin A rich food in the past 24 h§	Maternal report	72/111 (65%)	92/120 (77%)	95/134 (71%)	128/155 (83%)	NA	NA	..	220/275 (80%)	167/245 (68%)	0·002
	SQ-LNS consumed in previous 24 h	Maternal report	NA	110/117 (94%)	NA	128/155 (87%)	NA	NA	..	238/265 (90%)	NA	..

NA=not applicable. Data are n (%), unless otherwise indicated. SOC=standard of care. IYCF=infant and young child feeding. WASH=water, sanitation, and hygiene. IYCF plus WASH=both IYCF plus WASH implemented together. SQ-LNS=small-quantity lipid-based nutrient supplement. VHW=village health worker.

* Combined WASH collapses the two WASH-containing groups (WASH and IYCF plus WASH); non-WASH collapses the two groups not including WASH (SOC and IYCF).

† Combined IYCF collapses the two IYCF-containing groups (IYCF and IYCF plus WASH); non-IYCF collapses the two groups not including IYCF (SOC and WASH).

‡ There were a maximum of 20 liquid soap deliveries, 15 WaterGuard deliveries, 13 SQ-LNS deliveries, 15 intervention modules.

§ Calculations exclude SQ-LNS consumption. p values adjusted for clustering effect. Depending on the variable type, xtgee, multinomial, ordinal regression models with robust variance estimation, and Somers' D for medians, were used for comparing groups while handling within-cluster correlation.

Reported open defecation among household members was virtually eliminated in the WASH compared with the non-WASH groups and the vast majority had a latrine with a well-trodden path that was not being used for storage and a hand-washing station with soap or rubbing agent and water (table 2). At 12 months, 85% of women in the WASH groups reported they usually treat their drinking water. However, too few samples of water were tested for free chlorine to objectively validate water chlorination; we suspect uptake was modest: of 160 12-month water samples from WASH households that were tested, only 92 (58%) had >0·1ppm free chlorine. Fewer mothers in WASH households compared with non-WASH households reported ever seeing their child ingest soil or chicken faeces (table 2). Breastfeeding rates at 12 months were very high and did not differ across groups (table 2). The vast majority of children in the IYCF groups had consumed SQ-LNS in the previous 24 h. Compared with infants in the non-IYCF groups, a higher proportion in the IYCF groups met minimum dietary diversity, and had consumed foods that were animal source, iron rich, and vitamin-A rich the day before (table 2).

At the primary endpoint, median age of infants was 18·0 (IQR 17·8–18·6) months and did not differ significantly across treatment groups. The IYCF intervention significantly increased length for age Z score and haemoglobin concentration at 18 months (table 3). Length for age Z score was 0·26 higher (95% CI 0·09–0·43) and haemoglobin 2·9 g/L higher (95% CI 0·90–4·90) among children who received IYCF compared with those who did not. For both primary outcomes, the whole population was shifted upwards; there was no evidence of a greater effect of the IYCF intervention on the lower tails of length for age Z score and haemoglobin distributions (appendix). The IYCF intervention reduced the number of stunted children from 165 (50%) of 329 in the non-IYCF groups to 136 (40%) of 336 in the IYCF groups (absolute reduction 10%, 95% CI 2–17; RR 0·82, 95% CI 0·69–0·98). These findings were very similar in fully adjusted analyses (table 4). 45 (14%) of 319 children in the non-IYCF groups were anaemic at 18 months, compared with 24 (7%) of 329 in the IYCF groups (absolute difference 7%, 95% CI 2–12; RR 0·52, 95% CI 0·34–0·79). Results were attenuated in fully adjusted analyses, but comparisons between groups might have been limited by small numbers of events (table 4).

**Table 3:** Effect of WASH and IYCF interventions on primary and secondary continuous outcomes at 18 months of age among children born to HIV-positive mothers

		**Effects by group**		**Main effects combining groups**						
		**N**	**Mean (SD)**	Treatment group	N	Mean (SD)	**Unadjusted difference (95% CI)**	**p value**	Adjusted difference (95% CI)*	**p value**
**Primary outcomes**										
**LAZ**										
	Standard of care	145	−2·00 (1·20)	No IYCF	329	−1·99 (1·13)	Ref	..	Ref	..
	IYCF	146	−1·73 (1·10)	IYCF	336	−1·73 (1·12)	0·26 (0·09 to 0.43)	0·003	0·23 (0·10 to 0·37)	0·001
	WASH	184	−1·97 (1·08)	No WASH	291	−1·87 (1·16)	Ref	..	Ref	..
	IYCF plus WASH	190	−1·73 (1·14)	WASH	374	−1·85 (1·12)	0·01 (−0·16 to 0·18)	0·90	0·07 (−0·08 to 0·22)	0·37
**Haemoglobin (g/L)**										
	Standard of care	141	116·8 (10·9)	No IYCF	319	116·6 (12·9)	Ref	..	Ref	..
	IYCF	146	118·5 (11·2)	IYCF	329	119·5 (11·7)	2·9 (0·90 to 4·90)	0·005	2·70 (0·60 to 4·80)	0·013
	WASH	178	116·5 (14·3)	No WASH	287	117·6 (11·1)	Ref	..	Ref	..
	IYCF plus WASH	183	120·3 (12·1)	WASH	361	118·4 (13·3)	0·70 (−1·20 to 2·70)	0·47	1·10 (−0·90 to 3·20)	0·27
**Secondary continuous outcomes**										
**WAZ**										
	Standard of care	146	−0·99 (1·10)	No IYCF	329	−0·96 (1·14)	Ref	..	Ref	..
	IYCF	147	−0·96 (1·00)	IYCF	336	−0·94 (1·06)	0·01 (−0·16 to 0·19)	0·87	0·02 (−0·17 to 0·15)	0·86
	WASH	183	−0·93 (1·17)	No WASH	293	−0·98 (1·05)	Ref	..	Ref	..
	IYCF plus WASH	189	−0·91 (1·10)	WASH	372	−0·92 (1·13)	0·06 (−0·11 to 0·24)	0·47	0.05 (−0·12 to 0·21)	0·57
**WHZ**										
	Standard of care	146	−0·05 (1·07)	No IYCF	327	−0·05 (1·10)	Ref	..	Ref	..
	IYCF	147	−0·16 (1·08)	IYCF	336	−0·13 (1·09)	−0·09 (−0·27 to 0·09)	0·35	−0·08 (−0·27 to 0·10)	0·37
	WASH	181	−0·04 (1·12)	No WASH	293	−0·10 (1·08)	Ref	..	Ref	..
	IYCF plus WASH	189	−0·11 (1·10)	WASH	370	−0·08 (1·11)	0·03 (−0·15 to 0·21)	0·71	−0·02 (−0·21 to 0·17)	0·83
**Mid-upper arm circumference for age Z score**										
	Standard of care	146	−0·20 (0·94)	No IYCF	328	−0·19 (0·88)	Ref	..	Ref	..
	IYCF	147	−0·20 (0·90)	IYCF	337	−0·15 (0·93)	0·03 (−0·10 to 0·17)	0·63	−0·02 (−0·16 to 0·12)	0·76
	WASH	182	−0·18 (0·83)	No WASH	293	−0·20 (0·92)	Ref	..	Ref	..
	IYCF and WASH	190	−0·12 (0·94)	WASH	372	−0·15 (0·89)	0·05 (−0·09 to 0·19)	0·50	0·06 (−0·08 to 0·21)	0·39
**Head circumference Z score**										
	Standard of care	146	−0·55 (1·08)	No IYCF	328	−0·54 (1·12)	Ref	..	Ref	..
	IYCF	147	−0·51 (1·09)	IYCF	336	−0·44 (1·12)	0·10 (−0·07 to 0·27)	0·24	0·08 (−0·08 to 0·25)	0·31
	WASH	182	−0·53 (1·15)	No WASH	293	−0·53 (1·09)	Ref	..	Ref	..
	IYCF and WASH	189	−0·38 (1·14)	WASH	371	−0·46 (1·14)	0·07 (−0·10 to 0·24)	0·42	0·14 (−0·03 to 0·31)	0·098

IYCF=infant and young child feeding; WASH=water, sanitation and hygiene; IYCF plus WASH=both IYCF plus WASH implemented together. LAZ=length for age Z score. WAZ=weight for age Z score. WHZ=weight for height Z score.

* Covariates included in adjusted analyses for LAZ and secondary growth outcomes were maternal height, maternal mid-upper arm circumference, marital status, maternal co-trimoxazole in pregnancy, low birthweight, infant sex, fieldworker, wealth quintile, household keeps livestock inside house, recruitment calendar period; covariates included in adjusted analyses for haemoglobin were maternal age, maternal haemoglobin, maternal employment, maternal ART in pregnancy, maternal co-trimoxazole in pregnancy, infant sex, fieldworker.

**Table 4:** Effect of WASH and IYCF interventions on secondary dichotomous outcomes at 18 months of age among children born to HIV-positive mothers

	**Effects by group**		**Main effects combining groups**						
	N	n (%)	Treatment group	N	n (%)	Unadjusted relative risk (95% CI)	**p value**	Adjusted relative risk (95% CI)	**p value**
**Stunting (LAZ <–2·0)**									
SOC	145	75 (52%)	No IYCF	329	165 (50%)	Ref	..	Ref	..
IYCF	146	59 (40%)	IYCF	336	136 (40%)	0·81 (0·68 to 0·97)	0·020	0·83 (0·71 to 0·99)	0·040
WASH	184	90 (49%)	No WASH	291	134 (46%)	Ref	..	Ref	..
IYCF plus WASH	190	77 (41%)	WASH	374	167 (45%)	0·97 (0·81 to 1·15)	0·70	0·95 (0·80 to 1·12)	0·52
**Severe stunting (LAZ <–3·0)**									
Standard of care	145	22 (15%)	No IYCF	329	51 (16%)	Ref	..	Ref	..
IYCF	146	19 (13%)	IYCF	336	42 (13%)	0·82 (0·55 to 1·23)	0·34	Insufficient sample	NA
WASH	184	29 (16%)	No WASH	291	41 (14%)	Ref	..	Ref	..
IYCF plus WASH	190	23 (12%)	WASH	374	52 (14%)	0·97 (0·65 to 1·44)	0·87	Insufficient sample	NA
**Anaemia (Haemoglobin <105 g/L)**									
Standard of care	141	17 (12%)	No IYCF	319	45 (14%)	Ref	..	Ref	..
IYCF	146	7 (5%)	IYCF	329	24 (7%)	0·52 (0·34 to 0·79)	0·002	0·95 (0·90 to 0·99)	0·033
WASH	178	28 (16%)	No WASH	287	24 (8%)	Ref	..	Ref	..
IYCF plus WASH	183	17 (9%)	WASH	361	45 (13%)	1·42 (0·89 to 2·27)	0·14	1·01 (0·96 to 1·07)	0·57
**Underweight (WAZ <–2·0)**									
Standard of care	146	24 (16%)	No IYCF	329	56 (17%)	Ref	..	Ref	..
IYCF	147	27 (18%)	IYCF	336	61 (18%)	1·07 (0·77 to 1·48)	0·70	Insufficient sample	NA
WASH	183	32 (18%)	No WASH	293	51 (17%)	Ref	..	Ref	..
IYCF plus WASH	189	34 (18%)	WASH	372	66 (18%)	1·03 (0·74 to 1·42)	0·88	Insufficient sample	NA
**Wasted (WHZ<–2·0)**									
Standard of care	146	6 (4%)	No IYCF	327	13 (4%)	Ref	..	Ref	..
IYCF	147	6 (4%)	IYCF	336	18 (5%)	1·30 (0·61 to 2·76)	0·49	Insufficient sample	NA
WASH	181	7 (4%)	No WASH	293	12 (4%)	Ref	..	Ref	..
IYCF plus WASH	189	12 (6%)	WASH	370	19 (5%)	1·20 (0·56 to 2·57)	0·64	Insufficient sample	NA

IYCF=infant and young child feeding; WASH=water, sanitation and hygiene; IYCF plus WASH=both IYCF plus WASH implemented together. LAZ=length for age Z score. WAZ=weight for age Z score. WHZ=weight for height Z score.

*Covariates included in adjusted analyses for secondary growth outcomes were maternal height, maternal mid-upper arm circumference, marital status, maternal co-trimoxazole in pregnancy, low birthweight, infant sex, fieldworker, wealth quintile, household keeps livestock inside house, recruitment calendar period; covariates included in adjusted analyses for secondary anaemia outcomes were maternal age, maternal haemoglobin, maternal employment, maternal ART in pregnancy, maternal co-trimoxazole in pregnancy, infant sex, fieldworker. Insufficient cases of severe anaemia to estimate relative risks.

The WASH intervention had no effect on length for age Z score or haemoglobin concentrations. No difference was seen in mean length for age Z score (0·01, 95% CI −0·16 to 0·18) and mean haemoglobin concentration (0·7 g/L, 95% CI −1·20 to 2·70) at 18 months between children who received WASH and those who did not; effects were similar in adjusted analyses (table 3). There was no significant effect of the WASH intervention on stunting or anaemia (table 4). There were 134 (46%) of 291 stunted children in the non-WASH groups compared with 167 (45%) of 374 in the WASH groups (absolute difference 1%, 95% CI −9 to 6; RR 0·97, 95% CI 0·81–1·15), and there were 24 (8%) of 287 anaemic children in the non-WASH groups compared with 45 (12%) of 361 in the WASH groups (absolute difference 4%, 95% CI −1 to 9; RR 1·42, 95% CI 0·89–2·27). Neither intervention had an effect on other measures of growth (ie, weight for age, weight for height, mid-upper arm circumference, head circumference, or underweight or wasting; Table 3, Table 4); in a post-hoc analysis there was no effect of either intervention on the composite outcome of stunted and wasted: 7 (2%) of 335 children in the IYCF groups were stunted and wasted compared to 8 (2%) of 326 in the non-IYCF groups (absolute difference 0%; 95% CI −3 to 2); and 9 (2%) of 370 children in the WASH groups were stunted and wasted compared to 6 (2%) of 291 in the non-WASH groups (absolute difference 0%; 95% CI −2 to 3).

There were no significant differences in 7-day prevalence of diarrhoea between groups at 12 months (appendix) or 18 months (table 5). The numbers of children with dysentery and acute respiratory infection were too few to compare. Cumulative mortality through 18 months was not significantly different between groups. In the standard of care group, 13 (8%) of 165 children died, compared with 6 (4%) of 156 in the IYCF group, 14 (7%) of 205 in the WASH group, and 15 (7%) of 207 in the IYCF plus WASH group. Adjusted analyses were not done owing to the small absolute numbers of cases.

**Table 5:** Effect of IYCF and WASH on diarrhoea, dysentery, acute respiratory infection, and mortality at 18 months

	**Effects by group**		**Main effects combining groups**				
	**N**	**n (%)**	Treatment group	N	n (%)	Unadjusted risk ratio*(95% CI)	**p value**
**Diarrhoea**							
Standard of care	145	9 (6%)	No IYCF	328	20 (6%)	Reference	..
IYCF	147	13 (9%)	IYCF	336	23 (7%)	1·12 (0·62–2·03)	0·72
WASH	183	11 (6%)	No WASH	292	22 (8%)	Reference	..
IYCF plus WASH	189	10 (5%)	WASH	372	21 (6%)	0·73 (0·40–1·33)	0·31
**Dysentery**							
Standard of care	145	2 (1%)	No IYCF	326	4 (1%)	Reference	..
IYCF	147	0	IYCF	336	0	Insufficient sample	NA
WASH	181	2 (1%)	No WASH	292	2 (1%)	Reference	..
IYCF plus WASH	189	0	WASH	370	2 (<1%)	Insufficient sample	NA
**Acute respiratory infection**							
Standard of care	145	0	No IYCF	327	2 (1%)	Reference	..
IYCF	146	1 (1%)	IYCF	335	4 (1%)	Insufficient sample	NA
WASH	182	2 (1%)	No WASH	291	1 (<1%)	Reference	..
IYCF plus WASH	189	3 (2%)	WASH	371	5 (1%)	Insufficient sample	NA
**Death**							
Standard of care	165	13 (8%)	No IYCF	370	27 (7%)	Reference	..
IYCF	156	6 (4%)	IYCF	363	21 (6%)	0·81 (0·44–1·49)	0·49
WASH	205	14 (7%)	No WASH	321	19 (6%)	Reference	..
IYCF plus WASH	207	15 (7%)	WASH	412	29 (7%)	1·26 (0·68–2·34)	0·47

IYCF=infant and young child feeding. WASH=water, sanitation, and hygiene. NA=not applicable. All outcomes apart from death were based on maternal 7-day recall. Diarrhoea was defined as passage of three or more loose or watery stools in a 24 h period. Dysentery was defined as passage of stool with blood or mucus. Acute respiratory infection was defined as fast or difficult breathing (ie, rapid breathing or chest retractions, or both).

* Adjusted analyses were not done owing to the small absolute numbers of cases.

In the prespecified per-protocol analysis restricted to those with high-fidelity delivery of trial interventions, effects of IYCF and WASH on growth were similar to the intention-to-treat findings, but there was also a significant effect of IYCF on mean head circumference for age Z score (0·20 [95% CI 0·01–0·39] higher in IYCF compared with non-IYCF groups, p=0·037; appendix). The effects of IYCF on haemoglobin concentration and anaemia were more pronounced in the per-protocol analysis than in the intention-to-treat analysis: haemoglobin concentration was 4·3 g/L (95% CI 2·0–6·5) higher in IYCF compared with non-IYCF groups, and there were fewer anaemic children in the IYCF groups (14 [6%] of 253) than in the non-IYCF groups (39 [17%] of 236; absolute difference 11%, 95% CI 5–17; RR 0·35, 95% CI 0·22–0·56). The WASH intervention significantly reduced 7-day prevalence of diarrhoea at the 18-month visit in the per-protocol analysis: 17 (8%) of 209 children in the non-WASH groups had diarrhoea, compared with 10 (3%) of 290 in the WASH groups (absolute difference 5%, 95% CI 0–9); RR 0·42, 95% CI 0·21–0·85), but had no effect at 12 months or on other measures of morbidity. In a preplanned subgroup analysis, infant sex did not modify the effects of IYCF or WASH on either primary outcome (interactions all p>0·10).

When the effects of the interventions were limited only to children confirmed as HIV-uninfected (ie, removing children who were HIV-positive and HIV-unknown at 18 months), the overall findings were similar (appendix).

Serious adverse events were similar across trial groups (table 6). There were no serious adverse events or adverse events related to the trial interventions.

**Table 6:** Cumulative distribution of serious adverse events among HIV-positive women and HIV-exposed infants by randomised trial arm

	**Standard of care**	**IYCF**	**WASH**	**IYCF plus WASH**
Miscarriages (n/N)	5/174 (3%)	13/182 (7%)	11/213 (5%)	5/215 (2%)
Stillbirths (n/N)	2/174 (1%)	13/186 (15%)	2/219 (1%)	4/219 (2%)
Neonatal deaths (<1month; n/N)	6/174 (3%)	3/186 (2%)	9/219 (4%)	9/219 (4%)
Infant deaths (n/N)	8/174 (5%)	4/186 (2%)	5/219 (2%)	7/219 (3%)
Maternal hospitalisation (n/N)	8/174 (5%)	15/182 (8%)	7/213 (3%)	22/215 (10%)
Infant hospitalisation (n/N)	2/174 (1%)	7/186 (4%)	5/219 (2%)	2/219 (1%)

## Discussion

We evaluated the individual and combined effects of IYCF and improved WASH on child linear growth and haemoglobin concentrations among HIV-exposed infants, in whom stunting and anaemia are common. HIV-exposed infants have an excess risk of stunting and therefore might particularly benefit from public health interventions aimed at promoting healthy growth. We found that IYCF had a significant but modest effect on linear growth, reducing stunting by almost 20%, whereas there were no clear benefits from the household WASH intervention. IYCF also increased haemoglobin and reduced the prevalence of anaemia, but neither intervention consistently affected morbidity or all-cause mortality. Overall, these findings are similar to those reported previously among HIV-unexposed children,^11^ and demonstrate that in settings of high antenatal HIV prevalence, IYCF interventions would have substantial benefits at scale, whereas integrating household-level elementary WASH interventions typical of those commonly available to rural populations in low-income and middle-income countries (ie, pit latrines, hand-washing stations not connected to a water source, and point-of-use drinking water with monthly behaviour-change communication) is unlikely to confer additional benefits on child growth or anaemia.

Linear growth benefits among HIV-exposed uninfected infants could be particularly important because of the high risk of stunting in this vulnerable population: in the non-IYCF groups, 50% of HIV-exposed uninfected infants were stunted by 18 months, despite high uptake of interventions to prevent mother-to-child transmission. Although the effect of IYCF on linear growth was modest (increased length for age Z score by 0·26), it exceeded the effect size observed in non-HIV-exposed children in SHINE^11^ (0·16 length for age Z score increase) and the average effect size (0·10 length for age Z score increase) reported in a recent meta-analysis of complementary feeding interventions among predominantly HIV-unexposed infants.^17^ There might be differences in household food security, dietary diversity, and caregiving practices between infants born to HIV-positive and HIV-negative mothers, such that HIV-exposed uninfected children showed particular benefits for linear growth from provision of additional calories, micronutrients, and education focused on diversification and calorie enrichment of infant diets. IYCF did not reduce the prevalence of underweight or wasting in HIV-exposed children. The effect of IYCF on haemoglobin was also modest (2·9 g/L gain at 18 months) but translated into a substantial reduction in the proportion of anaemic children at 18 months. HIV-exposed infants have a higher frequency of anaemia than do HIV-unexposed infants,^18^, ^19^ and might respond better to iron supplementation, although the causes of anaemia in HIV-exposed uninfected infants remain unclear. Notably, all the trial findings were similar after excluding HIV-infected children and children of unknown HIV status.

The WASH intervention had no effect on growth or anaemia and inconsistent effects on diarrhoea in HIV-exposed children. Our hypothesis was that WASH would reduce diarrhoea and prevent environmental enteric dysfunction, which would, in turn, reduce stunting by reducing malabsorption and chronic inflammation. We reasoned that HIV-exposed infants might be particularly vulnerable to environmental enteric dysfunction, owing to perturbed composition and function of the microbiota, which is vertically transmitted from an HIV-positive mother,^20^ and direct effects of HIV exposure during breastfeeding on the gut barrier and intestinal mucosal CD4 cells.^21^ However, we found no effect of the WASH intervention on linear growth, and inconsistent reductions in diarrhoea between the intention-to-treat and per-protocol populations. Uptake of the WASH intervention was high, as assessed by structured observations and self-report, although the intervention intensity might have been too low to modify household behaviours to the extent necessary to affect these health outcomes.^11^ We previously speculated that the elementary WASH interventions implemented in the SHINE trial might not have been effective enough to reduce highly contaminated environments. We believe that so-called transformative WASH is urgently required, which combines better tools, more intensive behaviour change, and strengthened governance for implementing these interventions.^11^ Ongoing laboratory studies will determine whether the WASH interventions affected any biomarkers of environmental enteric dysfunction.

The WASH Benefits trials in Kenya^22^ and Bangladesh^23^ have recently reported the effects of IYCF and WASH interventions on growth and anaemia. SHINE was purposefully aligned with WASH Benefits in design, but was the only trial carried out in a setting with high HIV prevalence (15% in SHINE, <6% in WASH Benefits Kenya,^22^ and <0·1% in WASH Benefits Bangladesh^24^); we therefore tested mothers and infants for HIV and stratified results by maternal HIV status. Findings on stunting and anaemia were similar across the three trials, despite the differences in context and populations: all three trials showed benefits of IYCF but none found an effect of the elementary household-level WASH interventions tested on stunting and anaemia. Findings to date show that the IYCF benefits might be greater in HIV-exposed infants than in HIV-unexposed infants, providing important evidence that an IYCF intervention is likely to be especially effective in populations with high antenatal HIV prevalence. However, elementary household-level WASH interventions—unaccompanied by investments in more intensive behaviour-change communication, efficacious technologies, and strengthened governance systems of financing, regulation, and management—are unlikely to reduce child stunting or anaemia.

Our trial had several strengths and limitations. We delivered household-level public health interventions with high fidelity of implementation and created substantial contrast in hardware, commodities and behaviours between groups. The level of input was higher than would be provided in a typical nutrition or WASH programme delivered to rural areas of low-income countries. We were unable to blind interventions to participants or fieldworkers because of the nature of the household inputs; however, investigators were masked during analysis and the consistency of findings across both primary outcomes suggests this factor is unlikely to have caused substantial ascertainment bias. Despite our use of constrained randomisation, which balanced clusters on a range of variables, there were some baseline differences between groups; however, we did adjusted analyses to accommodate for this imbalance. Adjustment mostly led to very similar findings, except for the reduction in anaemia, which was significantly attenuated in the fully adjusted analysis. The finding for anaemia might be at least partly explained by a relatively small absolute number of anaemia cases (table 4). We were unable to determine HIV status for all children at 18 months because of caregiver refusal for blood draws, insufficient sample volume, or discordant results; however, in sensitivity analyses that removed children with unknown or positive HIV status, the effect of the interventions was unchanged. The trial was conducted in a community with high, but not universal, uptake of interventions to prevent mother-to-child transmission; however, we intervened in the trial to promote exclusive breastfeeding to high levels,^25^ which might have reduced HIV transmission^26^ and improved infant health outcomes regardless of randomised interventions. Finally, the sample size for the trial was based on detecting a difference in length for age Z score among HIV-unexposed infant groups; we did not calculate a specific sample size for HIV-exposed infants. It is possible that the null effect of the WASH intervention was due to insufficient power to detect an effect; however, we think this is unlikely given that there was no evidence of a difference in length for age Z score between the WASH and non-WASH groups in the much larger sample of 3686 HIV-unexposed children in whom we have previously reported findings.^11^

In summary, a complementary feeding intervention that promoted behaviour change and provided a daily calorie and micronutrient supplement in the form of a lipid-based nutrient supplement improved linear growth and haemoglobin concentration in HIV-exposed children at 18 months old. A household-level WASH intervention providing elementary WASH tools and monthly behaviour-change communication had no evidence of an effect on growth or anaemia, and no consistent effect on diarrhoea. These findings are similar to those observed in the HIV-unexposed children enrolled in SHINE. These findings are important because stunting and anaemia are major causes of morbidity, mortality, and impaired neurodevelopment globally,^8^ and interventions that improve linear growth and haemoglobin might confer benefits across the life course. Although the absolute effect of the IYCF intervention on mean length for age Z score and haemoglobin was modest, it led to substantial reductions in stunting and anaemia by 18 months of age, which might have considerable public health benefit at scale. Overall, our findings highlight the need for greater coverage of IYCF interventions, particularly in areas of ongoing high antenatal HIV prevalence, where they might be particularly beneficial, and provide an urgent call for WASH interventions that are more efficacious.

For the **protocol and statistical analysis plan** see https://osf.io/w93hy

For **interactive tools** see https://osf.io/w93hy

For the **protocol and statistical analysis plan** see https://osf.io/w93hy

For the **Clinical Epidemiology Database Resources** see http://ClinEpiDB.org
