# Supplementary material for: Independent and combined effects of improved water, sanitation, and hygiene, and improved complementary feeding, on stunting and anaemia among HIV-exposed children in rural Zimbabwe: a cluster-randomised controlled trial
Source: Lancet Child Adolesc Health. 2019 Feb;3(2):77–90. doi: 10.1016/S2352-4642(18)30340-7 (PMC6472652; doi:10.1016/S2352-4642(18)30340-7)
Supplement: Supplementary appendix [file mmc1.pdf]

THE LANCET
Child & Adolescent Health

Supplementary appendix

This appendix formed part of the original submission and has been peer reviewed. We post it as supplied by the authors.

Supplement to: Prendergast AJ, Chasekwa B, Evans C, et al. Independent and combined effects of improved water, sanitation, and hygiene, and improved complementary feeding, on stunting and anaemia among HIV-exposed children in rural Zimbabwe: a cluster-randomised controlled trial. *Lancet Child Adolesc Health* 2018; published online Dec 17. http://dx.doi.org/10.1016/S2352-4642(18)30340-7.

APPENDIX

A cluster-randomized trial of improved complementary feeding and improved water, sanitation and hygiene on stunting and anemia among HIV-exposed children in rural Zimbabwe

Andrew J. Prendergast DPhil[1,2,3], Bernard Chasekwa MSc[1], Ceri Evans MRCPCH[1,2], Kuda Mutasa MPH[1], Mduduzi N. N. Mbuya PhD[1,4], Rebecca J. Stoltzfus PhD[5], Laura E. Smith PhD[1,6], Florence D. Majo RGN[1], Naume V. Tavengwa MSW[1], Batsirai Mutasa MBA[1], Goldberg T. Mangwadu MSc[7], Cynthia M. Chasokela PhD[7], Ancikaria Chigumira MSc[7], Lawrence H. Moulton PhD[3], Robert Ntozini MPH[1] and Jean H. Humphrey ScD[1,3] for the SHINE Trial Team[8]

[1]Zvitambo Institute for Maternal and Child Health Research, Harare, Zimbabwe
[2]Blizard Institute, Queen Mary University of London, London, UK.
[3]Department of International Health, Johns Hopkins Bloomberg School of Public Health, Baltimore MD, USA
[4]Global Alliance for Improved Nutrition, Washington, DC, USA
[5]Division of Nutritional Sciences, Cornell University, Ithaca, NY, USA
[6]Department of Epidemiology and Environmental Health, School of Public Health and Health Professions, University at Buffalo, Buffalo, NY, USA
[7]Ministry of Health and Child Care, Government of Zimbabwe, Harare, Zimbabwe
[8] Members of the SHINE Trial team are listed in:
https://academic.oup.com/cid/article/61/suppl_7/S685/358186

CONTENTS

Supplementary methods
- a) Randomisation procedure p2
- b) Changes in gestational age enrolment criteria p2
- c) Further details of interventions p2-5
- d) Details of anthropometry measurements p5
- e) Definition of per protocol analysis p5
- f) Length-for-age Z-scores and haemoglobin concentrations at 18 months of age p6

References p6

Supplementary Table 1: Baseline characteristics of women and infants who completed and defaulted the 12 month visit p7

Supplementary Table 2: Per protocol primary outcomes p8-9

Supplementary Table 3: Effects of WASH and IYCF interventions on primary and secondary outcomes at 18 months of age among HIV-exposed uninfected children……………………………..p10-11

Supplementary Table 4: Serious Adverse Events by arm…………………………………….p12

SUPPLEMENTARY METHODS

a) Randomisation procedure

A highly constrained randomisation technique was used to allocate clusters (stratified by district) to treatments. We randomly selected 1000 allocations from among 5000 computer-generated allocations that balanced the 4 treatment arms on 14 parameters without pre-specified bounds. From the 1000, we randomly selected 10 for a public randomisation ceremony; Figure S1. Each randomisation scheme divided the randomisation units into 4 groups of approximately 53 units. Each scheme's corresponding colour-coded map was printed on a separate sheet and displayed at a public forum attended by all elected councillors from the study area, District and Provincial Administrators, and Ministry of Health and Child Care authorities. In their presence, 10 plastic balls (numbered 1-10) were placed in an opaque sack. A community representative selected one ball from the sack, thereby identifying which of the 10 numbered allocations would be used. Then, four balls (labelled A, B, C, and D) were placed in one sack, and four balls (labelled with the 4 treatment arms) were placed in a second sack. Representatives drew a ball from the first sack and a ball from the second sack, pairing a group of clusters with one of the four treatment arms, thereby mimicking a widely known World Cup draw procedure. This was repeated twice more to pair the next two groups of clusters with two more treatment arms. The remaining balls formed the final pairing. This second stage was included to provide an additional assurance of impartiality/randomness and a further opportunity for participation of the community leadership.

b) Changes in gestational age enrolment criteria

The goal at the start of the trial was to recruit women between 10-14 weeks gestation, so women more than 14 weeks gestation were excluded. However, this cut-off was liberalised three times over the life of the trial to maximise recruitment because many women were excluded due to pregnancy that was >14 gestational weeks at screening. The upper limit of gestational age was therefore increased to 18 weeks (August 22, 2013), 24 weeks (January 3, 2014), and any time prior to parturition (October 20, 2014), through trial protocol amendments.

c) Further details of interventions

Standard of Care (SOC) Intervention: Village Health Workers were trained through the Ministry of Health and Child Care curriculum, which instructs VHWs to visit pregnant women and infants frequently, although the precise content or purpose of each visit is not specified. Consequently the SHINE SOC intervention was designed to standardize the number of visits (3 antenatal and 12 postnatal visits) and the content of primary health care messages across treatment arms. Four of these visits promoted exclusive breastfeeding (EBF) from birth to 6 months using modules designed to overcome contextual barriers identified in formative work. Other SOC modules include prevention of mother-to-child HIV transmission (PMTCT), antenatal care, hospital-based delivery, family planning and immunizations.

WASH Intervention: Within 6 weeks of enrollment (~20 weeks gestation) into the WASH and WASH+IYCF arms of the trial, a Blair Ventilated Improved Pit (VIP) Latrine was constructed at the participant's household and two 'Tippy Tap' hand-washing stations (locally manufactured, and adapting the model piloted by the Kenya WASH Benefits trial) were installed near the latrine and kitchen; Figure S2.

WASH Modules 1 (delivered at 24 gestational weeks) and 2 (32 gestational weeks) promoted safe disposal of faeces, and hand-washing with soap after faecal contact and before food preparation and eating, respectively. Our intention was for the baby to be born into a household in which latrine use and household hand-washing behaviours were normalised and habitual. WASH Module 3 (protecting babies from faecal ingestion during exploratory play) was delivered when the baby was 2 months old; a washable 2.8m x 3.0m locally manufactured mat and plastic play yard (North States, Minneapolis MN) were provided at 2 months and 6 months, respectively; Figure S3.

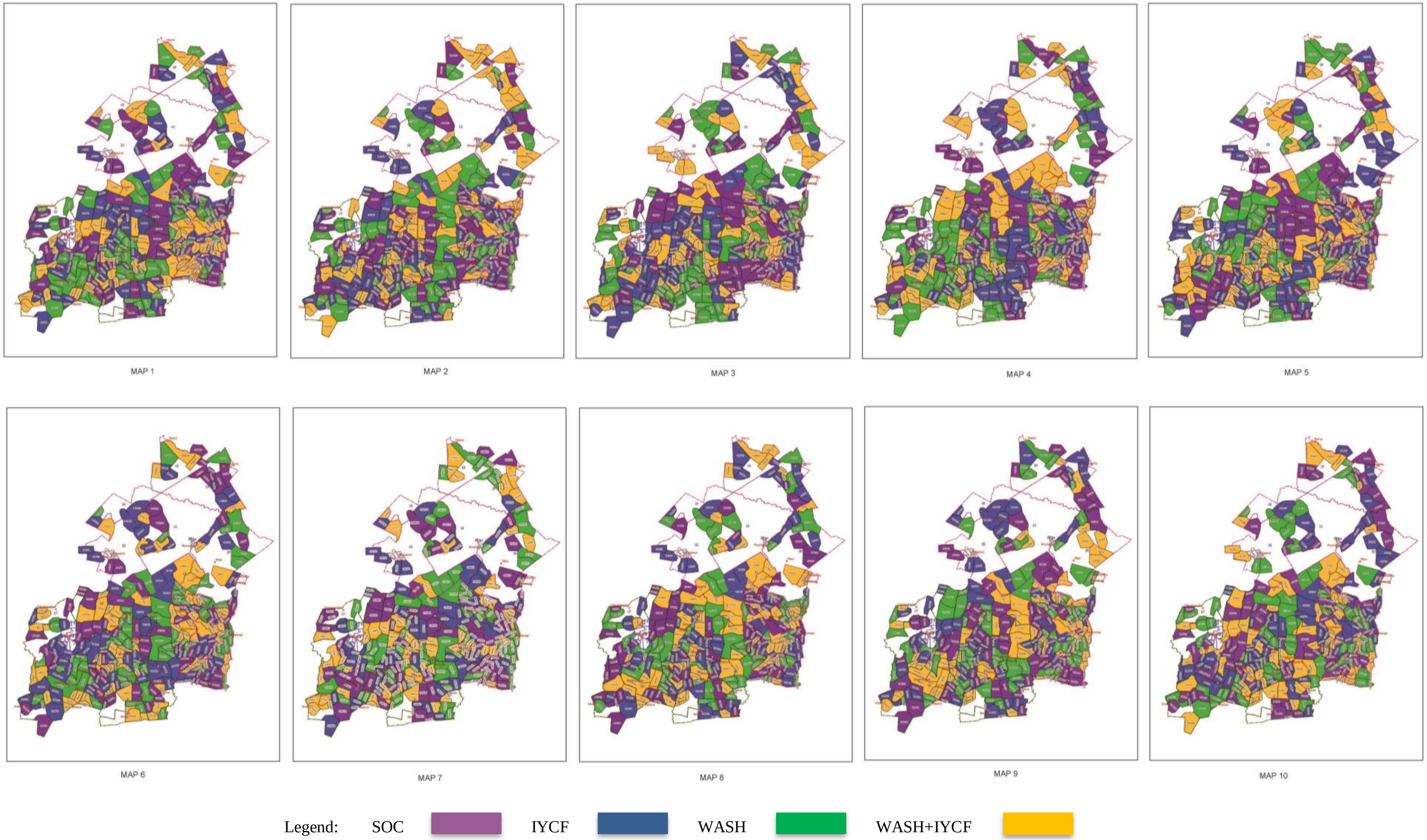

Figure S1. Maps of ten SHINE randomization schemes

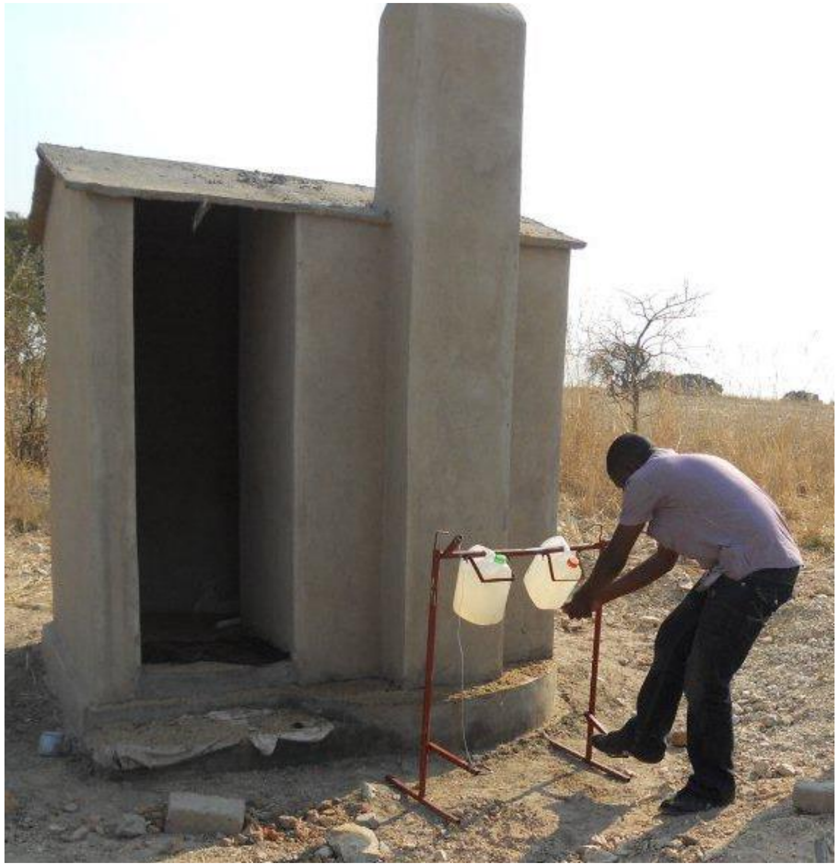

Figure S2: Blair VIP latrine and Tippy Tap

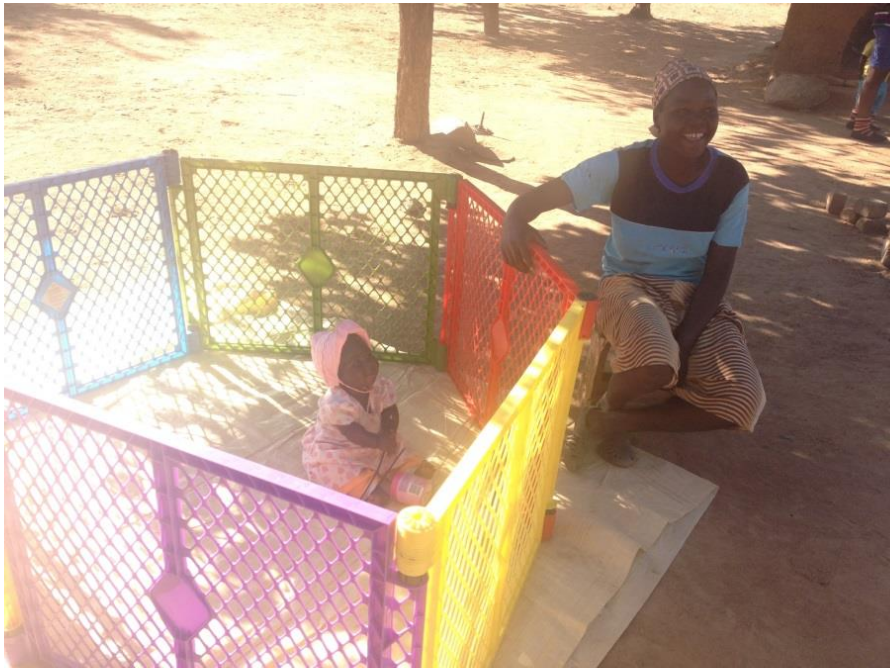

Figure S3: SHINE mat and play yard

WASH Module 4 (treat all drinking water given to babies after 6 months of EBF) was delivered at 4 months of age, along with point-of-use chlorination (WaterGuard: a dilute sodium hypochlorite solution, manufactured locally by Nelspot). Liquid soap and Water Guard were regularly replenished from time of introduction (Module 2 and 5, respectively) until the infant was 18 months old. WASH Module 5, delivered at 5 months of age, stressed the importance of freshly preparing or fully reheating all foods fed to infants. A review module was delivered at 12 months.

IYCF Intervention: IYCF Module 1 (delivered at 5 months) linked good infant feeding to child growth, health, and intelligence. IYCF Module 2 (6 months) promoted feeding nutrient-dense food, including 20 g per day of the small-quantity lipid-based nutrient supplement (SQ-LNS 20g infants) developed by the International Lipid-Based Nutrients Supplements Project, with composition as previous described[1]. This SQ-LNS was provided monthly when the baby was 6 to 18 months of age. Module 3 (7 months) was a participatory cooking demonstration in which any available household food was prepared and fed to the baby, stressing three messages from formative research: 1) an infant can eat any food that an adult eats; 2) food should be ground so that the infant can swallow and digest it; 3) food that is locally available is important for the infant. Module 4 (8 months) promoted responsive feeding during illness, Module 5 (9 months) promoted diet diversity, and a review module was delivered at 12 months.

d) Details of anthropometry measurements

Anthropometry was undertaken by trained nurses according to Standard Operating Procedures following one week of specific residential training, which formed part of their 14-week training course undertaken prior to the start of the trial. Recumbent infant length was measured to the nearest 0.1cm using a Seca 417 infantometer (Weigh & Measure LLC., Olney, MD, USA); at the 18-month endpoint visit, length was measured three times and the median value used in analysis. Infant weight was measured to the nearest 10g using a Tanita BD-590 infant scale (Weigh & Measure LLC., Olney, MD, USA). Mid-upper arm circumference was measured on the left side to the nearest 0.1cm using a multi-purpose ShorrTape (Weigh & Measure LLC., Olney, MD, USA). Head circumference was measured to the nearest 0.1cm using a multi-purpose ShorrTape (Weigh & Measure LLC., Olney, MD, USA). Nurses underwent 6-monthly standardisation exercises, in which the same ten mothers and ten children were measured by each nurse, and values compared to a gold standard anthropometrist. A coefficient of reliability (R) value against the gold standard anthropometrist was calculated and nurses with R values <0.95 for intra- and inter-observer accuracy were given further training and additional supervision in the field. Once the nurse supervisor was satisfied that anthropometry technique had improved, the nurse resumed independent measurements. Supervisors observed every nurse conducting a research visit at least quarterly and conducted spot checks with study participants to evaluate performance.

e) Definition of per protocol analysis

Secondary modified per protocol analyses were conducted with these restrictions:

For the IYCF + WASH group:
Higher Fidelity: received all 10 core modules
Lower Fidelity: received less than 10 core modules

For the SOC, IYCF, and WASH groups:
Higher Fidelity: received all modules scheduled for delivery at the same time points when the 10 core IYCF and WASH modules were delivered.

Lower Fidelity: received less than all modules scheduled for delivery at the same time points when the 10 core IYCF and WASH modules were delivered.

f) Impact of the IYCF intervention on length-for-age Z-scores and haemoglobin concentrations at 18 months of age among HIV-exposed children.

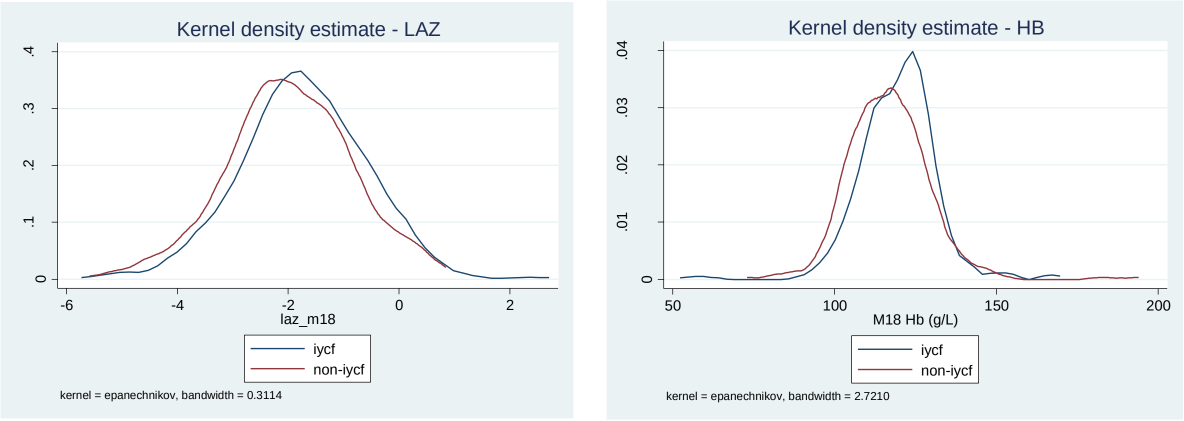

(a) (b)

Figure S4: (a) LAZ: length-for-age Z score (b) HB: Haemoglobin.

References

1. Arimond M, Zeilani M, Jungjohann S, Brown KH, Ashorn P, Allen LH, Dewey KG. Considerations in developing lipid-based nutrient supplements for prevention of undernutrition: experience from the International Lipid-Based Nutrient Supplements (iLiNS) Project. *Maternal and Child Nutrition* 2015; 11 (Suppl 4), 31-61.

Supplementary Table 1. Baseline maternal, household, and infant characteristics of HIV-positive mothers and their live born infants who defaulted and completed the 12 month visit.

Baseline characteristics[1]	Defaulted 12-month Visit	Completed 12-month Visit	p value
Mothers, N	136	521	
Infants, N	136	532	
Mothers completing baseline visit	134	515	
**Household characteristics**			
Size; median (IQR)	4 (3 - 6)	4 (3 - 6)	0.787
Wealth Quintile[2]			
Lowest	43/132 (33%)	124/512 (24%)	
Second	26/132 (20%)	127/512 (25%)	
Middle	26/132 (20%)	100/512 (20%)	0.254
Fourth	16/132 (12%)	79/512 (15%)	
Highest	16% (16%16%)	82/512 (16%)	
Electricity			
Power grid	4/132 (3%)	14/513 (3%)	0.850
Other power:			
Generator	5/132 (4%)	11/513 (2%)	
Solar	81/132 (61%)	337/513 (66%)	0.427
No electricity	46/132 (35%)	165/513 (32%)	
Sanitation			
Household members who openly defecate (all)	349/595 (59%)	1160/2203 (53%)	0.372
Any latrine at household	48/130 (37%)	167/504 (33%)	0.389
Improved latrine at household	41/129 (32%)	146/504 (29%)	0.492
Improved latrine with well-trodden path	33/129 (26%)	125/504 (25%)	0.835
Improved latrine with well-trodden path and not shared	29/125 (23%)	108/490 (22%)	0.752
Water			
Main source of household drinking water is improved	71/129 (55%)	310/504 (62%)	0.176
Treat drinking water to make it safer	16/129 (12%)	61/497 (12%)	0.968
One-way walk time to fetch water, minutes; median (IQR)	10 (5 - 30)	10 (5 - 20)	0.059
Per capita water volume collected past 24 h, L; mean (SD)	9.0 (5.8)	9.5 (7.1)	<0.001
Hygiene			
Hand-washing station at household	16/124 (13%)	48/471 (10%)	0.454
Hand-washing station with water	2/123 (2%)	13/467 (3%)	0.470
Hand-washing station with water and rubbing agent	0/123 (0%)	1/467 (0.2%)	<0.001
Improved floor[3]	56/131 (43%)	250/504 (50%)	0.168
Number of chickens; median (IQR)	4 (0 - 7)	5 (2 - 10)	0.025
Livestock in home	38/130 (29%)	184/508 (36%)	0.098
Feces observed in yard	40/128 (32%)	144/505 (29%)	0.514
Diet quality and food security			
Household meets minimum Diet Diversity Score[4]	43/114 (38%)	173/440 (39%)	0.744

Coping strategies Index[5]; median (IQR)	3 (0 - 13)	2 (0 - 9)	0.178
**Maternal characteristics**			
Age, years; mean (SD)	27 (6)	30 (6)	<0.001
Height, cm; mean (SD)	160 (11)	160 (6)	<0.001
MUAC, cm; mean (SD)	26 (3)	26 (3)	<0.001
S. haematobium, microscopy positive	17/125 (14%)	43/491 (9%)	<0.001
Completed schooling, years; mean (SD)	9.0 (2.6)	9.2 (2.1)	0.014
Parity; median (IQR)	2 (1 - 3)	2 (1 - 3)	<0.001
Married	115/129 (89%)	465/487 (95%)	0.019
Employed	16/132 (12%)	42/513 (8%)	<0.001
Religion:			0.003
Apostolic	55/131 (42%)	238/492 (48%)	
Other Christian	53/131 (40%)	214/492 (44%)	0.003
Other non-Christian religion	23/131 (18%)	40/492 (8%)	
**Infant characteristics**			
Female	75/136 (55%)	261/532 (49%)	0.209
Birth weight, kg; mean (SD)	3.0 (0.5)	3.0 (0.5)	<0.001
Birth weight <2500 g	17/114 (15%)	55/497 (11%)	0.315
Institutional delivery	91/121 (75%)	421/486 (87%)	0.001
Vaginal delivery	111/123 (90%)	458/494 (93%)	0.001

[1] Maternal and household baseline data collected about 2 weeks after consent (~14 weeks gestation). Baseline for infants was at birth. Values are %, unless noted.

[2] Chasekwa B, Maluccio JA, Ntozini R, et al. Measuring wealth in rural communities: Lessons from the Sanitation, Hygiene, Infant Nutrition Efficacy (SHINE) trial. *PLOS One* 2018, in press.

[3] Improved floor defined as concrete, brick, cement, or tile. Unimproved floor defined as mud, earth, sand, or dung.

[4] FAO, FHI 360. Minimum Dietary Diversity for Women: A Guide for Measurement. Rome: FAO. 2016.

[5] Maxwell D, Watkins B, Wheeler R, Collins G. The Coping Strategy Index: A tool for rapid measurement of household food security and the impact of food aid programs in humanitarian emergencies. CARE and WFP, Nairobi. 2003.

Supplementary Table 2. Effect of WASH and IYCF interventions on primary and secondary outcomes at 18 months of age in those with high fidelity delivery of interventions

Primary outcomes	Effects by arm			Main effects combining arms				
							Unadjusted	
	Treatment group	N	Mean (SD)	Treatment group	N	Mean (SD)	Difference (95% CI)	p
Length for age Z score	SOC	93	-1.93 (1.14)	IYCF: no	244	-1.94 (1.08)	0.00 (ref)	
	IYCF+SOC	117	-1.65 (1.17)	IYCF: yes	258	-1.66 (1.13)	0.28 (0.09, 0.47)	0.004
	WASH+SOC	151	-1.94 (1.04)	WASH: no	210	-1.78 (1.16)	0.00 (ref)	
	IYCF+WASH+SOC	141	-1.67 (1.11)	WASH: yes	292	-1.81 (1.08)	-0.02 (-0.21, 0.18)	0.876
Hemoglobin (g/dL)	SOC	89	115.8 (10.6)	IYCF: no	236	116.2 (13.6)	0.0 (ref)	
	IYCF+SOC	117	118.9 (11.6)	IYCF: yes	253	120.4 (11.4)	4.3 2.0, 6.5)	<0.001
	WASH+SOC	147	116.5 (15.2)	WASH: no	206	117.6 (11.3)	0.0 (ref)	
	IYCF+WASH+SOC	136	121.6 (11.1)	WASH: yes	283	119.0 (13.6)	1.8 (-0.4, 3.9)	0.109

Secondary dichotomous outcomes	Effects by arm			Main effects combining arms				
							Unadjusted	
	Treatment group	N	n (%)	Treatment group	N	n (%)	Relative risk (95% CI)	p
Stunting (LAZ<-2.0)	SOC	93	46 (49.5)	IYCF: no	244	120 (49.2)	1.00 (ref)	
	IYCF+SOC	117	46 (39.3)	IYCF: yes	258	101 (39.2)	0.80 (0.65, 0.97)	0.027
	WASH+SOC	151	74 (49.0)	WASH: no	210	92 (43.8)	1.00 (ref)	
	IYCF+WASH+SOC	141	55 (39.0)	WASH: yes	292	129 (44.2)	0.99 (0.81, 1.21)	0.917
Severe stunting (LAZ<-3.0)	SOC	93	12 (12.9)	IYCF: no	244	34 (13.9)	1.00 (ref)	
	IYCF+SOC	117	15 (12.8)	IYCF: yes	258	31 (12.0)	0.87 (0.53, 1.43)	0.582
	WASH+SOC	151	22 (14.6)	WASH: no	210	27 (12.9)	1.00 (ref)	
	IYCF+WASH+SOC	141	16 (11.4)	WASH: yes	292	38 (13.0)	0.95 (0.57, 1.56)	0.828
Anemia (Hb <105 g/L)	SOC	89	13 (14.6)	IYCF: no	236	39 (16.5)	1.00 (ref)	
	IYCF+SOC	117	6 (5.1)	IYCF: yes	253	14 (5.5)	0.35 (0.22, 0.56)	<0.001
	WASH+SOC	147	26 (17.7)	WASH: no	206	19 (9.2)	1.00 (ref)	
	IYCF+WASH+SOC	136	8 (5.9)	WASH: yes	283	34 (12.0)	1.13 (0.71, 1.80)	0.604
Severe anemia (Hb <70 g/L)	SOC	89	0 (0.0)	IYCF: no	236	0 (0.0)	1.00 (ref)	
	IYCF+SOC	117	1 (0.8)	IYCF: yes	253	1 (0.4)	Insufficient sample	NA
	WASH+SOC	147	0 (0.0)	WASH: no	206	1 (0.5)	1.00 (ref)	
	IYCF+WASH+SOC	136	0 (0.0)	WASH: yes	283	0 (0.0)	Insufficient sample	NA
Underweight (WAZ<-2.0)	SOC	93	16 (17.2)	IYCF: no	243	40 (16.5)	1.00 (ref)	
	IYCF+SOC	118	19 (16.1)	IYCF: yes	258	41 (15.9)	0.96 (0.65, 1.43)	0.849
	WASH+SOC	150	24 (16.0)	WASH: no	211	35 (16.6)	1.00 (ref)	
	IYCF+WASH+SOC	140	22 (15.7)	WASH: yes	290	46 (15.9)	0.96 (0.64, 1.44)	0.837

**Wasted (WHZ<-2.0)**	SOC	93	3 (3.2)	IYCF: no	241	9 (3.8)	1.00 (ref)	
	IYCF+SOC	118	4 (3.4)	IYCF: yes	258	13 (5.0)	1.41 (0.62, 3.21)	0.411
	WASH+SOC	148	6 (4.1)	WASH: no	211	7 (3.3)	1.00 (ref)	
	IYCF+WASH+SOC	131	9 (6.4)	WASH: yes	288	15 (5.2)	1.64 (0.69, 3.89)	0.260
**Diarrhea at 12 months**	SOC	81	8 (9.9)	IYCF: no	203	21 (10.3)	0.0 (ref)	
	IYCF+SOC	104	7 (6.7)	IYCF: yes	237	25 (10.5)	1.03 (0.60, 1.77)	0.910
	WASH+SOC	122	13 (10.7)	WASH: no	185	15 (8.1)	0.0 (ref)	
	IYCF+WASH+SOC	133	18 (13.5)	WASH: yes	255	31 (12.2)	1.53 (0.87, 2.67)	0.139
**Diarrhea at 18 months**	SOC	91	7 (7.7)	IYCF: no	241	15 (6.2)	0.0 (ref)	
	IYCF+SOC	118	10 (8.5)	IYCF: yes	258	12 (4.6)	0.69 (0.36, 1.33)	0.267
	WASH+SOC	150	8 (5.3)	WASH: no	209	17 (8.1)	0.0 (ref)	
	IYCF+WASH+SOC	140	2 (1.4)	WASH: yes	290	10 (3.4)	0.42 (0.21, 0.85)	0.015
**Secondary continuous outcomes**	**Effects by arm**			**Main effects combining arms**				
							**Unadjusted**	
	**Treatment Group**	**N**	**Mean (SD)**	**Treatment Group**	**N**	**Mean (SD)**	**Difference (95% CI)**	**p**
**WAZ**	SOC	93	-1.04 (1.04)	IYCF: no	243	-0.97 (1.11)	0.0 (ref)	
	IYCF+SOC	118	-0.88 (1.02)	IYCF: yes	258	-0.85 (1.06)	0.11 (-0.09, 0.31)	0.264
	WASH+SOC	150	-0.92 (1.15)	WASH: no	211	-0.95 (1.03)	0.0 (ref)	
	IYCF+WASH+SOC	140	-0.83 (1.10)	WASH: yes	290	-0.88 (1.12)	0.09 (-0.11, 0.29)	0.370
**WHZ**	SOC	93	-0.15 (1.06)	IYCF: no	241	-0.11 (1.10)	0.0 (ref)	
	IYCF+SOC	118	-0.10 (1.08)	IYCF: yes	258	-0.07 (1.08)	0.04 (-0.15, 0.24)	0.651
	WASH+SOC	148	-0.08 (1.11)	WASH: no	211	-0.12 (1.07)	0.0 (ref)	
	IYCF+WASH+SOC	140	-0.04 (1.08)	WASH: yes	288	-0.06 (1.09)	0.06 (-0.13, 0.25)	0.554
**MUACZ**	SOC	93	-0.31 (0.89)	IYCF: no	242	-0.21 (0.85)	0.0 (ref)	
	IYCF+SOC	118	-0.11 (0.92)	IYCF: yes	259	-0.07 (0.93)	0.15 (-0.02, 0.31)	0.076
	WASH+SOC	149	-0.16 (0.83)	WASH: no	211	-0.20 (0.91)	0.0 (ref)	
	IYCF+WASH+SOC	141	-0.03 (0.95)	WASH: yes	290	-0.10 (0.89)	0.12 (-0.04, 0.29)	0.147
**Head circumference Z**	SOC	93	-0.64 (1.00)	IYCF: no	243	-0.57 (1.13)	0.0 (ref)	
	IYCF+SOC	118	-0.44 (1.11)	IYCF: yes	259	-0.38 (1.09)	0.20 (0.01, 0.39)	0.037
	WASH+SOC	150	-0.52 (1.20)	WASH: no	211	-0.53 (1.06)	0.0 (ref)	
	IYCF+WASH+SOC	141	-0.32 (1.07)	WASH: yes	291	-0.43 (1.14)	0.11 (-0.08, 0.30)	0.244

Supplementary Table 3. Effects of WASH and IYCF interventions on primary and secondary outcomes at 18 months of age among HIV-exposed uninfected children

Primary outcomes	Effects by arm			Main effects combining arms						
	Treatment group	N	Mean (SD)	Treatment group	N	Mean (SD)	Unadjusted		Adjusted[1]	
							Difference (95% CI)	p	Difference (95% CI)	p
Length for age Z score	SOC	134	-1.96 (1.13)	IYCF: no	297	-1.97 (1.07)	0.00 (ref)		0.00 (ref)	
	IYCF	134	-1.74 (1.11)	IYCF: yes	294	-1.74 (1.11)	0.22 (0.04, 0.40)	0.017	0.20 (0.05, 0.36)	0.010
	WASH	163	-1.97 (1.01)	WASH: no	268	-1.85 (1.12)	0.00 (ref)		0.00 (ref)	
	IYCF+WASH	160	-1.75 (1.12)	WASH: yes	323	-1.86 (1.07)	-0.01 (-0.19, 0.17)	0.94	0.09 (-0.07, 0.25)	0.286
Haemoglobin (g/L)	SOC	131	116.9 (10.7)	IYCF: no	291	117.3 (12.6)	0.0 (ref)		0.00 (ref)	
	IYCF	135	119.1 (11.1)	IYCF: yes	291	120.1 (10.6)	2.8 (0.8, 4.7)	0.005	3.2 1.2, 5.2)	0.002
	WASH	160	117.7 (14.0)	WASH: no	266	118.0 (10.9)	0.0 (ref)		0.00 (ref)	
	IYCF+WASH	156	120.9 (10.2)	WASH: yes	316	119.3 (12.4)	1.2 (-0.8, 3.1)	0.24	0.9 (-1.2, 2.9)	0.406

Secondary dichotomous outcomes	Effects by arm			Main effects combining arms						
	Treatment group	N	n (%)	Treatment group	N	n (%)	Unadjusted		Adjusted[1]	
							Relative risk (95% CI)	p	Relative risk (95% CI)	p
Stunting (LAZ<-2.0)	SOC	134	69 (51.5)	IYCF: no	297	150 (50.5)	1.00 (ref)		1.00 (ref)	
	IYCF	134	55 (41.7)	IYCF: yes	294	121 (41.2)	0.82 (0.68, 0.99)	0.041	0.84 (0.69, 1.01)	0.064
	WASH	163	81 (49.7)	WASH: no	268	124 (46.3)	1.00 (ref)		1.00 (ref)	
	IYCF+WASH	160	66 (41.3)	WASH: yes	323	147 (45.5)	0.97 (0.81 1.17)	0.79	0.93 (0.78, 1.12)	0.46
Severe stunting (LAZ<-3.0)	SOC	134	19 (14.2)	IYCF: no	297	42 (14.1)	1.00 (ref)		1.00 (ref)	
	IYCF	134	19 (14.2)	IYCF: yes	294	37 (12.6)	0.90 (0.59, 1.37)	0.63	Insufficient sample	NA
	WASH	163	23 (14.1)	WASH: no	268	38 (14.2)	1.00 (ref)		1.00 (ref)	
	IYCF+WASH	160	18 (11.3)	WASH: yes	323	41 (12.7)	0.88 (0.58, 1.34)	0.57	Insufficient sample	NA
Anaemia (Hb <10.5 g/dL)	SOC	131	15 (11.5)	IYCF: no	291	35 (12.0)	1.00 (ref)		1.00 (ref)	
	IYCF	135	4 (3.0)	IYCF: yes	291	15 (5.2)	0.43 (0.26, 0.73)	0.002	0.94 (0.90, 0.98)	0.006
	WASH	160	20 (12.5)	WASH: no	266	19 (7.1)	1.00 (ref)		1.00 (ref)	
	IYCF+WASH	156	11 (7.1)	WASH: yes	316	31 (9.8)	1.22 (0.69, 2.13)	0.50	1.03 (0.98, 1.08)	0.25
Underweight (WAZ<-2.0)	SOC	135	22 (16.3)	IYCF: no	297	48 (16.2)	1.00 (ref)		1.00 (ref)	
	IYCF	135	26 (19.3)	IYCF: yes	295	55 (18.6)	1.15 (0.81, 1.63)	0.42	Insufficient sample	NA
	WASH	162	26 (16.1)	WASH: no	270	48 (17.8)	1.00 (ref)		1.00 (ref)	
	IYCF+WASH	160	29 (18.1)	WASH: yes	322	55 (17.1)	0.97 (0.69, 1.37)	0.85	Insufficient sample	NA
Wasted (WHZ<-2.0)	SOC	135	6 (4.4)	IYCF: no	295	12 (4.1)	1.00 (ref)		1.00 (ref)	
	IYCF	135	5 (3.7)	IYCF: yes	295	16 (5.4)	1.33 (0.63, 2.79)	0.45	Insufficient sample	NA
	WASH	160	6 (3.8)	WASH: no	270	11 (4.1)	1.00 (ref)		1.00 (ref)	
	IYCF+WASH	160	11 (6.9)	WASH: yes	320	17 (5.3)	1.30 (0.62, 2.75)	0.49	Insufficient sample	NA

Secondary continuous outcomes	Effects by arm			Main effects combining arms						
							Unadjusted		Adjusted[1]	
	Treatment Group	N	Mean (SD)	Treatment Group	N	Mean (SD)	Difference (95% CI)	p	Difference (95% CI)	p
WAZ	SOC	135	-0.99 (1.09)	IYCF: no	297	-0.93 (1.12)	0.0 (ref)		0.0 (ref)	
	IYCF	135	-0.97 (1.01)	IYCF: yes	295	-0.95 (1.04)	-0.03 (-0.21, 0.15)	0.76	-0.03 (-0.19, 0.14)	0.75
	WASH	162	-0.87 (1.15)	WASH: no	270	-0.98 (1.05)	0.0 (ref)		0.0 (ref)	
	IYCF+WASH	160	-0.94 (1.07)	WASH: yes	322	-0.90 (1.11)	0.08 (-0.10, 0.26)	0.36	0.06 (-0.12, 0.23)	0.51
WHZ	SOC	135	-0.08 (1.08)	IYCF: no	295	-0.03 (1.10)	0.0 (ref)		0.0 (ref)	
	IYCF	135	-0.16 (1.09)	IYCF: yes	295	-0.14 (1.08)	-0.11 (-0.30, 0.07)	0.22	-0.09 (-0.27, 0.09)	0.35
	WASH	160	0.02 (1.12)	WASH: no	270	-0.12 (1.08)	0.0 (ref)		0.0 (ref)	
	IYCF+WASH	160	-0.13 (1.08)	WASH: yes	320	-0.05 (1.10)	0.07 (-0.11, 0.25)	0.44	-0.02 (-0.21, 0.18)	0.87
MUACZ	SOC	135	-0.20 (0.97)	IYCF: no	297	-0.17 (0.88)	0.0 (ref)		0.0 (ref)	
	IYCF	135	-0.20 (0.92)	IYCF: yes	295	-0.17 (0.92)	-0.00 (-0.15, 0.15)	0.99	-0.03 (-0.17, 0.12)	0.72
	WASH	162	-0.13 (0.80)	WASH: no	270	-0.20 (0.94)	0.0 (ref)		0.0 (ref)	
	IYCF+WASH	160	-0.13 (0.93)	WASH: yes	322	-0.13 0.87)	0.07 (-0.08, 0.22)	0.35	0.07 (-0.08, 0.23)	0.37
Head circumference Z	SOC	135	-0.53 (1.07)	IYCF: no	296	-0.53 (1.12)	0.0 (ref)		0.0 (ref)	
	IYCF	135	-0.51 (1.09)	IYCF: yes	294	-0.47 (1.10)	0.06 (-0.12, 0.24)	0.52	0.07 (-0.11, 0.25)	0.44
	WASH	161	-0.53 (1.17)	WASH: no	270	-0.52 (1.08)	0.0 (ref)		0.0 (ref)	
	IYCF+WASH	159	-0.43 (1.11)	WASH: yes	320	-0.48 (1.14)	0.04 (-0.14, 0.22)	0.66	0.13 (-0.05, 0.32)	0.17

[1]Covariates included in adjusted analyses for LAZ and secondary growth outcomes were maternal height, maternal MUAC, marital status, maternal co-trimoxazole in pregnancy, low birth weight, infant gender, fieldworker, wealth quintile, household keeps livestock inside house, recruitment calendar period

[1]Covariates included in adjusted analyses for haemoglobin and secondary anaemia outcomes were maternal age, maternal haemoglobin, maternal employment, maternal ART in pregnancy, maternal co-trimoxazole in pregnancy, infant gender, fieldworker

Insufficient cases of severe anaemia to estimate relative risks

Supplementary Table 4. Effect of IYCF and WASH on diarrhoea, dysentery, and acute respiratory infection at 12 months

	N	Prevalence (%)		Main effect combining groups			Unadjusted risk ratio* (95% CI)	p value
				Treatment group	N	Prevalence (%)		
**Diarrhoea**								
SOC	112	11·6		No IYCF	246	11·4	Ref	¨
IYCF	121	7·4		IYCF	283	10·6	0·91 (0·59–1·40)	0·66
WASH	134	11·2		No WASH	233	9·4	Ref	¨
IYCF plus WASH	162	13·0		WASH	296	12·2	1·32 (0·84–2·08)	0·23
**Dysentery**								
SOC	112	0 (0%)		No IYCF	245	0·4	Ref	¨
IYCF	121	0 (0%)		IYCF	283	0 (0%)	Insufficient sample	NA
WASH	133	0·8		No WASH	233	0 (0%)	Ref	¨
IYCF plus WASH	162	0 (0%)		WASH	295	0·3	Insufficient sample	NA
**Acute respiratory infection**								
SOC	112	0 (0%)		No IYCF	246	0·8	Ref	¨
IYCF	121	0 (0%)		IYCF	283	0 (0%)	Insufficient sample	NA
WASH	134	1·5		No WASH	233	0 (0%)	Ref	¨
IYCF plus WASH	162	0 (0%)		WASH	296	0·7	Insufficient sample	NA

SOC=standard of care. IYCF=infant and young child feeding. WASH=water, sanitation, and hygiene. Ref=reference. NA=not applicable. Based on maternal 7-day recall. Diarrhoea was defined as passage of three or more loose or watery stools in a 24 h period. Dysentery was defined as passage of stool with blood or mucus. Acute respiratory infection was defined as fast or difficult breathing (ie, rapid breathing or chest retractions, or both). *Adjusted analyses were not undertaken done owing to the small absolute numbers of cases.
